# Genetic variation in the endocannabinoid system and response to Cognitive Behavior Therapy for child anxiety disorders

**DOI:** 10.1002/ajmg.b.32467

**Published:** 2016-06-27

**Authors:** Kathryn J. Lester, Jonathan R. I. Coleman, Susanna Roberts, Robert Keers, Gerome Breen, Susan Bögels, Cathy Creswell, Jennifer L. Hudson, Anna McKinnon, Maaike Nauta, Ronald M. Rapee, Silvia Schneider, Wendy K. Silverman, Mikael Thastum, Polly Waite, Gro Janne H. Wergeland, Thalia C. Eley

**Affiliations:** ^1^ School of PsychologyUniversity of SussexBrightonUK; ^2^ King's College LondonMRC SocialGenetic and Developmental Psychiatry (SGDP) CentreInstitute of Psychiatry, Psychology and NeuroscienceLondonUK; ^3^ National Institute for Health Research Biomedical Research CentreSouth London and Maudsley National Health Service TrustBeckenhamUK; ^4^ Research Institute Child Development and EducationUniversity of AmsterdamAmsterdamThe Netherlands; ^5^ School of Psychology and Clinical Language SciencesUniversity of ReadingReadingUK; ^6^ Department of PsychologyCentre for Emotional HealthMacquarie UniversitySydneyAustralia; ^7^ MRC Cognition and Brain Sciences UnitCambridgeUK; ^8^ Brain and Mind Research InstituteUniversity of SydneySydneyAustralia; ^9^ Department of Clinical Psychology and Experimental PsychopathologyUniversity of GroningenGroningenThe Netherlands; ^10^ Department of PsychologyRuhr‐Universität BochumBochumGermany; ^11^ Yale University School of MedicineChild Study CenterNew HavenConnecticut; ^12^ Department of Psychology and Behavioural SciencesAarhus UniversityAarhusDenmark; ^13^ Department of Child and Adolescent PsychiatryHaukeland University HospitalBergenNorway

**Keywords:** anxiety, endocannabinoids, fear extinction, Cognitive Behavior Therapy, children

## Abstract

Extinction learning is an important mechanism in the successful psychological treatment of anxiety. Individual differences in response and relapse following Cognitive Behavior Therapy may in part be explained by variability in the ease with which fears are extinguished or the vulnerability of these fears to re‐emerge. Given the role of the endocannabinoid system in fear extinction, this study investigates whether genetic variation in the endocannabinoid system explains individual differences in response to CBT. Children (N = 1,309) with a primary anxiety disorder diagnosis were recruited. We investigated the relationship between variation in the CNR1, CNR2, and FAAH genes and change in primary anxiety disorder severity between pre‐ and post‐treatment and during the follow‐up period in the full sample and a subset with fear‐based anxiety disorder diagnoses. Change in symptom severity during active treatment was nominally associated (*P *< 0.05) with two SNPs. During the follow‐up period, five SNPs were nominally associated with a poorer treatment response (rs806365 [CNR1]; rs2501431 [CNR2]; rs2070956 [CNR2]; rs7769940 [CNR1]; rs2209172 [FAAH]) and one with a more favorable response (rs6928813 [CNR1]). Within the fear‐based subset, the effect of rs806365 survived multiple testing corrections (*P *< 0.0016). We found very limited evidence for an association between variants in endocannabinoid system genes and treatment response once multiple testing corrections were applied. Larger, more homogenous cohorts are needed to allow the identification of variants of small but statistically significant effect and to estimate effect sizes for these variants with greater precision in order to determine their potential clinical utility. © 2016 The Authors. *American Journal of Medical Genetics Part B: Neuropsychiatric Genetics* Published by Wiley Periodicals, Inc.

## INTRODUCTION

Childhood anxiety disorders are very common [Kessler et al., 2005] and are associated with a wide range of impairments [Kim‐Cohen et al., 2003; Erath et al., 2007; Asendorpf et al., 2008]. Response to Cognitive Behavior Therapy (CBT) varies substantially between patients [James et al., 2013]. Identifying predictors of response is important given the potential for clinicians to identify children and adolescents at risk for poorer outcomes before treatment begins and to help inform the development of more efficacious therapies. Recent years have seen a growing interest in the genetic prediction of response to psychological therapy, a field known as therapygenetics [Lester and Eley, 2013]. Yet to receive attention is the endocannabinoid (ECB) system, despite a growing literature implicating endocannabinoids in the pathogenesis of anxiety and fear, fear extinction, and emotional processing [Lafenetre et al., 2007; Hillard et al., 2012; Ruehle et al., 2012; Mechoulam and Parker, 2013].

Extinction learning is assumed to be an important component of CBT, in which individuals are repeatedly exposed to their feared object, situation, or anxiety‐provoking thought in the absence of any aversive consequences. Over successive exposures, the patient learns that their feared object is not predictive of an aversive outcome and anxiety is reduced [Craske et al., 2014]. However, extinguished fears are vulnerable to recovery and can re‐emerge with the passage of time, which creates limitations on the potential durability and effectiveness of CBT [Craske et al., 2008; Arch and Craske, 2009]. This is because extinction is a new learning process that involves the encoding of a new competing memory, but which does not replace the original fear memory, leaving it potentially ready to re‐emerge [Bouton, 2002]. A feature of anxiety disorders both in adult and child samples is their tendency to recur even following initially successful treatment with relapse rates reported to approximate 20–30% in child and adolescent samples [Gearing et al., 2013; Piacentini et al., 2014]. Surprisingly, little is known about predictors of relapse. One possibility is that individual differences in the ease with which fears are extinguished and/or vulnerability of extinguished fears to re‐emerge may in part explain inter‐individual variation in initial response and risk of relapse following CBT.

The ECB system comprises of cannabinoid receptors (CB1 and CB2), the endogenous endocannabinoids (anandamide [AEA] and 2‐arachidonoylglycerol [2‐AG]), and the catabolic enzymes for endocannabinoid degradation (fatty acid amide hydrolase [FAAH] for AEA and monoacylglycerol lipase [MAGL] for 2‐AG). Considerable research supports the hypothesis that endogenous endocannabinoid signaling regulates anxiety. There is also suggestive evidence that targeting components of the ECB system via activation of CB1 receptors or by manipulating FAAH activity may produce anxiolytic effects [Kathuria et al., 2003; Lafenetre et al., 2007; Gunduz‐Cinar et al., 2013]. Pertinent to our understanding of the factors influencing treatment response is research in adults demonstrating the role of the ECB system in fear extinction [Gunduz‐Cinar et al., 2013]. Failure to effectively extinguish fear when cues that previously predicted threat are no longer present can lead to the maintenance of fear and has been proposed as an important mechanism in the etiology of anxiety disorders [Hofmann, 2008].

Animal research has shown that genetic deletion and pharmacological blockade of CB1 receptors impedes extinction [Marsicano et al., 2002; Lafenetre et al., 2007]. In contrast, enhancing cannabinoid neurotransmission using either anandamide reuptake inhibitors, which alter FAAH activity or direct CB1 agonists facilitates fear extinction [Chhatwal et al., 2005; Pamplona et al., 2006; Bitencourt et al., 2008]. The ECB system may be particularly important for the consolidation and retention of extinction memories [Suzuki et al., 2008] thus attenuating the spontaneous recovery of conditioned fear responding. Two studies, one [Rabinak et al., 2013], which administered tetrahydrocannabinol (THC) pre‐extinction, and a second [Das et al., 2013] that administered cannabidiol after extinction learning found consolidation of extinction learning to be enhanced in human participants. However, a third study [Klumpers et al., 2012], which also administered THC did not detect an effect of THC on consolidation of fear extinction. Several studies have also shown that administration of cannabinoid system modulators, such as THC, modulates the neural substrates (amygdala, ventromedial prefrontal cortex, hippocampus) involved in extinction learning, extinction memory recall [Rabinak et al., 2014], and the processing of emotional stimuli [Phan et al., 2008; Fusar‐Poli et al., 2009; Bossong et al., 2013]. Given these findings, cannabinoid‐based pharmacotherapy and augmentation of existing treatments has been proposed as a promising avenue for the development of novel treatments for anxiety disorders [Domschke and Zwanzger, 2008; Graham and Milad, 2011; Fitzgerald et al., 2014], although as of yet the evidence for efficacy remains unclear [Whiting et al., 2015].

Recent research has investigated the effects of genetic variability in human endocannabinoid signaling for fear extinction. Numerous single nucleotide polymorphisms (SNPs) have been identified in CNR1 and CNR2, the genes that encode for cannabinoid receptor 1 and 2, respectively and in FAAH, the gene that encodes for the FAAH protein, the primary regulator of AEA signaling in the brain [Cravatt et al., 2001]. Variation in rs2180619, a SNP in the promoter region of CNR1 has been associated with fear extinction. G allele carriers demonstrated robust extinction of fear evidenced by a reduction in fear‐potentiated startle relative to AA homozygote carriers who failed to extinguish fear [Heitland et al., 2012]. A small number of variants in CNR1 (e.g., rs1049353; rs806368) and CNR2 (rs2501431) have been investigated in the context of emotional processing of socially relevant stimuli [Chakrabarti et al., 2006; Domschke et al., 2008] and in predicting antidepressant treatment response in patients with Major Depression [Domschke et al., 2008; Mitjans et al., 2012, 2013].

Research in mice has shown that FAAH inhibitors facilitate extinction by augmenting AEA signaling in the amygdala. Similarly, healthy carriers of the low‐expressing A allele at rs324420, which leads to reduced expression of FAAH and elevated levels of AEA showed reduced amygdala activity [Hariri et al., 2009]. Furthermore, low expressing A allele carriers showed more rapid habituation of amygdala responses to threatening stimuli relative to CC homozygotes [Gunduz‐Cinar et al., 2013]. They also reported lower scores on a personality measure of stress reactivity [Gunduz‐Cinar et al., 2013]. A recent study showed persuasive convergent effects of FAAH variation in both humans and mice. Human A allele carriers showed enhanced fear extinction indexed by reduced skin conductance response to the extinguished cue and lower levels of trait anxiety. Mice carrying the A allele demonstrated reduced freezing behavior on presentation of the extinguished cue and decreased anxiety in response to two measures of anxiety‐like behaviors that involved placing the mice in conflict situations (elevated plus maze test and novelty induced hypophagia test) [Dincheva et al., 2015]. These findings suggest that variation in FAAH may be an important moderator of anxiety‐related behaviors and is a plausible candidate for involvement in determining for whom psychological treatments involving exposure components will be most effective.

In the current study, we tested the association between polymorphisms of the CNR1, CNR2, and FAAH genes and response to CBT in children and adolescents with an anxiety disorder diagnosis. To our knowledge, this is the first study to investigate genetic variation in the endocannabinoid system and response to a psychological treatment. We began by testing our hypotheses in a large sample of children (N = 1,309) experiencing the full range of anxiety disorder diagnoses and who had received a course of CBT in order to maximize power to detect genetic effects. However, one possibility is that extinction learning may be implicated more or less in the mechanisms of treatments for different disorders. For example, extinction learning may be of greater relevance for the successful treatment of predominantly fear based disorders such as specific phobias and to a lesser extent for distress based disorders like generalized anxiety disorder [Borkovec and Ruscio, 2001]. Thus, in secondary analyses, we tested our hypotheses in a subset of the sample (N = 749) that had received a fear‐based anxiety disorder diagnosis (e.g., specific phobia, social phobia, separation anxiety disorder, panic disorder). Theses analyses were informed by research using genetic and phenotypic data to determine the structure of psychopathology [Lahey et al., 2004; Clark and Watson, 2006; Watson et al., 2008] and which suggests that emotional disorders can be decomposed into distress disorders (e.g., major depression, generalized anxiety disorder, posttraumatic stress disorder); fear disorders (e.g., phobias, panic disorder); and the bipolar disorders [Watson et al., 2008].

We tested two hypotheses. Firstly, that genetic variation in CNR1, CNR2, and FAAH would be significantly associated with change in symptom severity from baseline to post‐treatment reflecting the influence of genetic variation in the ECB system during the active treatment period. One possibility is that any effect of ECB genes on early symptom change may reflect the role of the ECB system in the extinction of fear. Second, we examined whether ECB genetic variation was associated with change in symptom severity from post‐treatment to follow‐up reflecting the influence of ECB genetic variation on maintenance of treatment gains. While for some, this will reflect a period in which they continue to consolidate the gains made during treatment, for others this may reflect a period in which they begin experiencing a relapse of symptoms. One possibility is that any effect of ECB genes on symptom change and specifically the continuance of treatment gains during the follow‐up period may reflect the role of the ECB system in the maintenance of extinction memories.

## MATERIALS AND METHODS

### Participants

Participants were recruited for the Genes for Treatment Study (G × T) study, a multi‐site international collaboration designed to identify clinical, demographic and genetic predictors of outcome following CBT for anxiety disorders in children and adolescents [Hudson et al., 2015]. The sample comprised 1,309 individuals for whom treatment response data was available at the post and/or follow‐up time points and genotype data was available for one or more SNPs. Participants were 5–17 years of age (89.6% aged 5–12 years, mean age: 9.81 years, 52% female) and met DSM‐IV criteria for primary diagnosis of an anxiety disorder. Exclusion criteria comprised significant physical or intellectual impairment, psychoses, and concurrent treatment. Participants completed a course of CBT as part of a trial or as treatment as usual at one of eleven sites: Sydney, Australia (n = 641); Reading and Oxford, UK (n = 302 and n = 15); Aarhus, Denmark (n = 123); Bergen, Norway (n = 39); Groningen, The Netherlands (n = 36); Bochum, Germany (n = 52); Florida, US (n = 38); Basel, Switzerland (n = 47), Cambridge, UK (n = 12); and Amsterdam, The Netherlands (n = 4). All treatments were manualized and treatment protocols across sites were comparable for core elements of CBT including teaching of coping skills, cognitive restructuring, and exposure. Three broad groups of treatment modality were given: individual CBT (27.4%), group based CBT (52.8%), and parent‐supported guided self‐help CBT (19.9%). Follow‐up data was collected at three (n = 231), 6 (n = 675), or 12‐months (n = 250) after cessation of treatment. Further sample characteristics for the full sample are given in Table I and site‐specific trial information is given in the supplementary information accompanying this article. Sample characteristics for the subset with a fear‐based diagnosis (excluding Generalized Anxiety Disorder (GAD), Obsessive Compulsive Disorder (OCD), Post Traumatic Stress Disorder (PTSD), Anxiety Disorders Not Otherwise Specified (ADNOS)) are given in Table SI in the supplementary materials.

**Table I:** Sample Characteristics by Site for the Full Sample

Characteristic	Sydney	Reading	Aarhus	Bergen	Bochum	Basel	Groningen	Oxford	Florida	Cambridge	Amsterdam	Total
N	641	302	123	39	52	47	36	15	38	12	4	1,309
Gender Female n (%)	317 (49.5)	165 (54.6)	70 (56.9)	24 (61.5)	30 (57.7)	26 (55.3)	17 (47.2)	9 (60.0)	18 (47.4)	8 (66.7)	0 (0)	684 (52.3)
Age: mean (SD)	9.41 (1.91)	9.59 (1.71)	11.02 (2.40)	11.46 (1.96)	11.15 (2.58)	8.49 (2.07)	11.89 (3.11)	9.00 (1.60)	9.61 (2.26)	12.58 (2.83)	12.00 (1.83)	9.81 (2.16)
Severity primary diagnosis: mean (SD)	6.3 (0.88)	5.62 (0.79)	6.53 (1.22)	6.72 (1.28)	6.77 (1.13)	5.98 (0.77)	6.19 (0.95)	5.60 (0.91)	6.82 (1.14)	6.33 (1.15)	5.75 (1.71)	6.22 (1.00)
Primary diagnosis: n (%)												
GAD	339 (52.8)	93 (30.8)	31 (25.2)	10 (25.6)	5 (9.6)	0 (0)	7 (19.4)	1 (6.7)	8 (21.1)	0 (0)	0 (0)	494 (37.7)
SoAD	136 (21.2)	62 (20.5)	18 (14.6)	17 (43.6)	15 (28.8)	0 (0)	14 (38.9)	6 (40.0)	10 (26.3)	0 (0)	1 (25.0)	279 (21.3)
SP	51 (8.0)	49 (16.2)	19 (15.4)	0 (0)	17 (32.7)	0 (0)	6 (16.7)	1 (6.7)	5 (13.2)	0 (0)	1 (25.0)	149 (11.4)
SAD	74 (11.5)	77 (25.5)	37 (30.1)	12 (30.8)	13 (25.0)	47 (100)	6 (16.7)	6 (40.0)	10 (26.3)	0 (0)	2 (50.0)	284 (21.7)
Other AD^a^	41 (6.4)	21 (7.0)	18 (14.6)	0 (0)	2 (3.8)	0 (0)	3 (8.3)	1 (6.7)	5 (13.2)	12 (100)	0 (0)	103 (7.9)
CBT treatment: n (%)												
Individual based	20 (3.1)	128 (52.4)	2 (1.6)	22 (56.4)	52 (100)	47 (100)	36 (100)	0 (0)	38 (100)	12 (100)	1 (25.0)	358 (27.4)
Group‐based	550 (85.8)	0 (0)	121 (98.4)	17 (43.6)	0 (0)	0 (0)	0 (0)	0 (0)	0 (0)	0 (0)	3 (75.0)	691 (52.8)
Guided self‐help	71 (11.1)	174 (57.6)	0 (0)	0 (0)	0 (0)	0 (0)	0 (0)	15 (100)	0 (0)	0 (0)	0 (0)	260 (19.9)

^a^ Other anxiety disorders include Panic Disorder, Obsessive Compulsive Disorder, Post Traumatic Stress Disorder, Selective Mutism, and Anxiety Disorders Not Otherwise Specified.

### Measures

Diagnoses were made using the Anxiety Disorders Interview Schedule for DSM‐IV (ADIS‐IV‐C/P) [Silverman and Albano, 1996] at all sites except for Bochum and Basel where the German equivalent Kinder‐DIPS was used [Schneider et al., 2009]. Clinical Severity Ratings (CSRs) ranged from 0 to 8 and were based on composite parent and child reports (see Hudson et al., 2015, for further details). Treatment response was assessed as change in primary diagnosis severity from pre‐treatment to post‐treatment and from post‐treatment to follow‐up. A diagnosis was assigned when the child met diagnostic criteria and received a CSR of four or greater. Primary diagnoses included Generalized Anxiety Disorder (GAD; 37.7%), Separation Anxiety Disorder (SAD; 21.7%), Social Anxiety Disorder (21.3%), Specific Phobia (11.4%), or Panic Disorder, Obsessive Compulsive Disorder, Post Traumatic Stress Disorder, Selective Mutism,^1^ or Anxiety Disorders Not Otherwise Specified (other anxiety disorders; 7.9%).

### Genotyping

DNA was collected using buccal swabs or Oragene saliva samples (DNA Genotek, Ottawa, Canada). Buccal swab DNA was extracted using established procedures designed to maximize the purity and yield of the sample [Freeman et al., 2003]. DNA from saliva samples was extracted using Prep‐it.L2P according to the manufacturers protocol (DNA Genotek). Sample preparation prior to genotyping is described elsewhere [Coleman et al., 2016]. In brief, samples were subjected to ultrafiltration and resuspension to increase DNA concentration and included in genotyping if the resulting concentration exceeded 50 ng/ul.

Genotypes for seven CNR1 polymorphisms (rs2180619; rs1049353; rs806368; rs806371; rs806379; rs1535255; rs806369), one CNR2 polymorphism (rs2501431) and one FAAH polymorphism (rs324420) drawn from the candidate gene literature on fear extinction, emotional processing, and response to antidepressant treatment were genotyped by LGC Genomics (Hoddesdon, UK) using validated arrays with KASP technology or were obtained from the Illumina Core Exome‐12v1.0 microarray. Four additional markers, which were genotyped using both platforms showed an average of 98% consensus on genotype calls.

For the subset of the sample with array data (n = 980) additional genotypes were available for 123 CNR1 polymorphisms, 159 CNR2 polymorphisms, and 318 FAAH polymorphisms. Array data was included in all analyses to provide LD context for multiple testing corrections and to provide more accurate gene‐based tests of association.

Quality control and imputation procedures for those samples with microarray data are provided in full elsewhere [Coleman et al., 2016]. Briefly, common variants (minor allele frequency >5%) were included in the analyses if they were genotyped in >99% of samples and if they did not deviate substantially from Hardy–Weinberg equilibrium (HWE test *P*‐value > 10^−5^). SNPs were included if they could be imputed to the December 2013 release of the 1000 Genomes Project reference [1000GenomesConsortium, 2012] with >90% completeness, and an info metric of >0.8 (a value ranging between 0 and 1 which indicates the certainty with which the SNP has been imputed). Using these cut‐offs, data was available for 127 CNR1 SNPs, 160 CNR2 SNPs, and 318 FAAH SNPs. Gene coverage estimated using directly genotyped and imputed SNPs meeting criteria for inclusion was 11.5%, 19.8%, and 28.9% for CNR1, CNR2, and FAAH genes, respectively. For each gene, analyzed variants were entered as tagging SNPs in the Tagger utility of Haploview [Barrett et al., 2005]. All common variants (MAF ≧ 0.05) within and ±100 kb of the gene boundaries (as listed in HapMap release II + III) were in linkage disequilibrium (r^2 ^> 0.8) with at least one tagging SNP. This indicates good coverage of all linkage regions across the genes studied. To account for patterns of linkage disequilibrium (LD) between SNPs, LD based clumping was performed for each analysis to reduce the SNP set to a smaller number of clumps of correlated SNPs.

### Ethical Approval

Each site had trial‐specific Human Ethics and Biosafety Committee approval for the collection of biological samples with the research conducted in accordance with the Declaration of Helsinki. In all instances parents provided written informed consent, children assent. The storage and analysis of DNA was approved by the King's College London Psychiatry, Nursing and Midwifery Research Ethics Sub‐Committee.

### Analyses

Two outcome measures were considered in our primary analyses. First, the change in severity (CSR score) of the primary anxiety diagnosis from baseline to post‐treatment, reflecting the active treatment period. Second, the change in severity of the primary anxiety diagnosis from post‐treatment to follow‐up time points, reflecting a period of consolidation or risk for relapse.

Linear mixed effects models were performed to investigate the effect of ECB polymorphisms on change in severity (CSR score) of the primary anxiety disorder diagnosis. All genotypes were coded to reflect an additive model where −1, common homozygote; 0, heterozygote; and 1, rare homozygote. To make use of all available post baseline measurements and provide estimates in the presence of missing data, the effects of predictors of response were estimated using mixed models fitted with full maximum likelihood. All models included either the fixed effects of baseline severity (CSR of the primary anxiety disorder diagnosis at baseline, centred at the mean) or post‐treatment severity (CSR of the primary anxiety disorder diagnosis at post‐treatment, centred at the mean), age (centred at the mean), and gender. Analyses investigating post to follow‐up change also included the linear and quadratic effects of time to account for the curvilinear slope of treatment response across this period. All models included the random effects of individual to account for correlations between repeated measures from the same individual. We also included a higher order random effect of trial to account for between trial differences in outcome. As each trial was conducted at a single site, this random effect also accounted for between‐site differences.

In all analyses, the beta values of variables predicting a more favorable response to treatment or continued gains during the follow‐up period (i.e., greater reduction in severity) are negative, while variables predicting a less favorable response are positive. Analyses were performed in STATA version 12.0.

All analyses (baseline to post‐treatment change, post‐treatment to follow‐up change, fear‐based diagnoses subset ((N = 749), gene‐based association tests) consider data from all available SNPs including both directly genotyped SNPs available on the entire sample (N = 1,309) and the additional SNPs available for the subset of the sample with array data (n = 980). N's are given for each sentinel SNP in the corresponding table for each analysis.

Results from the initial association analyses were clumped based on patterns of LD according to *P*‐value using PLINK [Purcell et al., 2007], thus reducing the SNP set to a smaller number of correlated SNPs. Each independent clump was represented by a sentinel SNP (that with the lowest *P*‐value in the clump), and contained all SNPs in linkage disequilibrium with the sentinel (R^2 ^> 0.25, within 250 kb of the sentinel). To correct for multiple testing, revised significance thresholds were calculated based on the number of independent clumps identified for each analysis.

Gene‐based tests for association with response were performed using VEGAS modified to use the hg19 genome build [Liu et al., 2010]. Gene boundaries were defined as the longest transcript of the gene listed in the UCSC Genome Browser and variants considered ±100 kb from each end. Linkage disequilibrium patterns were calculated from the genotyped data.

### Power Calculations

Power calculations indicated that with a sample size of 980, we had 80% power to detect a variant with a minor allele frequency of 0.05 capturing 1.6% of variance with a corrected alpha level of 0.017. For variants explaining 0.1%, 0.5%, and 1% of the variance we had 1.6%, 18%, and 50% power, respectively.

## RESULTS

Clinical outcomes in the full sample were comparable to previously reported estimates [Hudson et al., 2015, 2013; James et al., 2013]. Following treatment, 58% of the sample was free of their primary anxiety disorder diagnosis with this rate rising to 67% by follow‐up. Symptom severity reduced significantly between baseline (6.22) and post‐treatment (2.97, t(1256) = 54.57, *P* < 0.0001) and post‐treatment and follow‐up time points (2.42, t(1256) = 9.40, *P *< 0.0001). We initially explored the effects of clinical (baseline severity; primary diagnosis; treatment type) and demographic factors (age; gender) on change in symptom severity between baseline and post‐treatment. Findings were broadly similar to those reported for the full sample [Hudson et al., 2015]. Individuals with Social Anxiety Disorder, Specific Phobias, or Separation Anxiety Disorder showed a significantly poorer response to treatment compared to those with Generalized Anxiety Disorder (β = 0.24, *P *< 0.0001; β = 0.11, *P *= 0.005, and β = 0.06, *P *= 0.044, respectively. Higher severity at baseline was associated with significantly poorer response to treatment (β = 0.30, *P* < 0.0001). However, treatment response did not differ according to sex, age, or treatment type (all *P* values > 0.05). For change in symptom severity between post‐treatment and follow‐up time points, individuals with Specific Phobias showed a significantly poorer treatment response compared to those with Generalized Anxiety Disorder (β = 0.15, *P *= 0.011). Higher severity at post‐treatment was also associated with significantly poorer response during the follow‐up period (β = 0.19, *P* < 0 .0001). Response during the follow‐up period did not differ according to sex, age, or treatment type (all *P* values > 0.05). A highly similar pattern of results was observed when the sample was restricted to those with a fear‐based anxiety disorder diagnosis only.

### Change in Symptom Severity From Baseline to Post‐Treatment: Analyses Using the Entire Sample (N = 1,309)

Thirty independent clumps were identified based on patterns of LD and were used to calculate adjusted *P* values for multiple testing corrections (*P* < 0.0017). Each independent clump was represented by a sentinel SNP (that with the lowest *P*‐value in the clump), and contained all SNPs in linkage disequilibrium with the sentinel (R^2^ > 0.25, within 250 kb of the sentinel). Two independent clumps were nominally associated with response (*P* < 0.05). An increasing number of copies of the minor allele of rs12133557 was associated with a more favorable treatment response (i.e., greater reductions in severity) across the treatment period. In contrast, the minor allele of the sentinel SNP rs6454676 (with this clump including the directly genotyped rs1535255) was associated with a poorer treatment response (i.e., smaller reductions in severity or an increase in severity associated with increasing number of copies of the minor allele, see Table II). However, neither of these effects survived multiple testing corrections at *P *< 0.0017. The remaining SNPs all had *P* values exceeding 0.05. Analyses restricted to a subset that identified as having four White European grandparents (n = 916) are available in the supplementary materials. Gene based association tests were non‐significant (CNR1: *P *= 0.172; CNR2: *P *= 0.202; FAAH: *P *= 0.846).

**Table II:** Independent Clumps Nominally Associated (*P *< 0.05) With Treatment Response Between (a) Baseline and Post‐Treatment and (b) Post‐Treatment and Follow‐Up

(a) Change in symptom severity from baseline to post‐treatment									
Sentinel SNP	Gene	Clump BP	Minor allele	MAF	Info	β	95% CI	*P*	n^a^
rs12133557	*CNR2*	24191219–24223859	T	0.098	0.978	−0.07	−0.14 to −0.01	0.020	925
rs6454676	*CNR1*	88860482–88885426	A	0.104	0.977	0.07	0.002–0.13	0.042	926

(b) Change in symptom severity from post‐treatment to follow‐up									
Sentinel SNP	Gene	Clump BP	Minor allele	MAF	Info	β	95% CI	*P*	n
rs806365	*CNR1*	88843390–88845949	T	0.408	Genotyped (microarray)	0.11	0.04–0.18	0.004	702
rs2501431	*CNR2*	24108683–24206032	G	0.423	Genotyped (LGC)	0.09	0.03–0.16	0.007	874
rs2070956	*CNR2*	24191219–24223859	C	0.101	0.995	0.14	0.02–0.26	0.021	698
rs6928813	*CNR1*	88860482–88885426	G	0.180	Genotyped (microarray)	−0.11	−0.20 to −0.01	0.033	702
rs7769940	*CNR1*	88947649–88973751	T	0.209	Genotyped (microarray)	0.10	0.01–0.19	0.034	702
rs2209172	*FAAH*	46938837–46978946	T	0.206	Genotyped (microarray)	0.09	0.00–0.18	0.044	702

All genotypes were coded to reflect an additive model where −1, common homozygote; 0, heterozygote; and 1, rare homozygote.

Regression weights (β) significantly less than 0 indicate that an increasing number of copies of the minor allele of the SNP was associated with a greater reduction in symptom severity across the active treatment or follow‐up period. Values significantly greater than 0 indicate that an increasing number of copies of the minor allele of the SNP was associated with a poorer reduction in symptom severity.

^a^ n reflects total number of cases included in regression analysis for the sentinel SNP.

### Change in Symptom Severity From Baseline to Post‐Treatment: Fear‐Based Anxiety Disorder Diagnosis Subset (N = 749)

Twenty‐nine independent clumps were identified based on patterns of LD and were used to calculate adjusted *P* values for multiple testing corrections (*p *< 0.0017). Two independent clumps were nominally associated with response (*P* < 0.05) with an increasing number of copies of the minor allele of the sentinel SNP rs6454676 (with this clump including the directly genotyped rs1535255) associated with a poorer treatment response (see Table III). The minor allele of rs12133557 was associated with a more favorable treatment response across the treatment period. However, neither of these effects survived multiple testing corrections. Gene based tests on this subset were non‐significant (CNR1: *P *= 0.129; CNR2: *P *= 0.148; FAAH: *P *= 0.694).

**Table III:** Independent Clumps Nominally Associated (*P *< 0.05) With Treatment Response Between (a) Baseline and Post‐Treatment and (b) Post‐Treatment and Follow‐Up in the Subset of the Sample With Fear‐Based Anxiety Disorder Diagnoses (n = 749)

(a) Change in symptom severity from baseline to post‐treatment									
Sentinel SNP	Gene	Clump BP	Minor allele	MAF	Info	β	95% CI	*P*	n^a^
rs12133557	*CNR2*	24191219–24223859	T	0.094	0.978	−0.11	−0.20 to −0.03	0.011	540
rs6454676	*CNR1*	88860482–88885426	A	0.108	0.977	0.09	0.005–0.17	0.038	539

(b) Change in symptom severity from post‐treatment to follow‐up									
Sentinel SNP	Gene	Clump BP	Minor allele	MAF	Info	β	95% CI	*P*	n
rs806365	*CNR1*	88843390–88845949	T	0.392	Genotyped (microarray)	0.17	0.07–0.27	**0.0011**	399
rs7769940	*CNR1*	88947649–88973751	T	0.216	Genotyped (microarray)	0.19	0.07–0.32	0.003	399
rs2501431	*CNR2*	24108683–24206032	G	0.448	Genotyped (LGC)	0.14	0.04–0.23	0.004	495

All genotypes were coded to reflect an additive model where −1, common homozygote; 0, heterozygote; and 1, rare homozygote.

Effects that survived multiple testing corrections are highlighted in bold.

Regression weights (β) significantly less than 0 indicate that an increasing number of copies of the minor allele of the SNP was associated with a greater reduction in symptom severity across the active treatment or follow‐up period. Values significantly greater than 0 indicate that an increasing number of copies of the minor allele of the SNP was associated with a poorer reduction in symptom severity.

^a^ n reflects total number of cases included in regression analysis for the sentinel SNP.

### Change in Symptom Severity From Post‐Treatment to Follow‐Up

Thirty independent clumps were identified and were used to calculate adjusted *P* values for multiple testing corrections (*P *< 0.0017). Of these, five independent clumps were associated with a poorer response (i.e., smaller reductions in severity or an increase in severity associated with an increasing number of copies of the minor allele) during the follow‐up period at a nominal *P*‐value of < 0.05 (sentinel SNPs: rs806365; rs2501431; rs2070956; rs7769940; rs2209172) while one independent clump (sentinel SNP: rs6928813) predicted a more favorable response (i.e., greater reductions in severity associated with increasing number of copies of minor allele). All clumps with *P *< 0.05 are displayed in Table II. However, none of the suggestively significant clumps survived multiple testing correction (*P *< 0.0017) with rs806365 having the lowest *P‐*value at *P *= 0.004. Analyses restricted to a subset that identified as having four White European grandparents are available in the supplementary materials. Gene based association tests on the full sample were all non‐significant (CNR1: *P *= 0.360; CNR2: *P *= 0.092; FAAH: *P *= 0.745).

### Change in Symptom Severity From Baseline to Follow‐Up: Fear‐Based Anxiety Disorder Diagnosis Subset

Thirty‐one independent clumps were identified and were used to calculate adjusted *P* values for multiple testing corrections (*P *< 0.0016). Of these, three independent clumps were associated with a poorer response (i.e., smaller reductions in severity or an increase in severity associated with increasing number of copies of the minor allele) during the follow‐up period at a nominal *P*‐value of <0.05 (sentinel SNPs: rs806365; rs7769940; rs2501431, see Table III). These same SNPs were nominally significant in the analyses using the entire dataset. However, the effects were stronger when examined in the subset of participants with fear‐based disorders, with the effect for rs806365 surviving multiple testing corrections (*P *= 0.0011). Gene based tests on this subset were non‐significant (CNR1: *P *= 0.620 CNR2: *P *= 0.053; FAAH: *P *= 0.335).

## DISCUSSION

Given the potential role of the ECB system in fear extinction and the maintenance of extinction memories, this study investigated whether genetic variation in the CNR1, CNR2, and FAAH genes was associated with response to CBT in children and adolescents with an anxiety disorder. In our analyses, two SNPs (rs12133557 and rs6454676) were nominally associated (*P* < 0.05) with change in symptom severity in both the entire sample and the subset with fear based diagnoses. An increasing number of copies of the minor allele of rs12133557 was associated with a more favorable response during the active treatment period. In contrast, an increasing number of copies of the minor allele of rs6454676 was associated with a poorer response during the active treatment period. However, these effects did not survive stringent multiple testing correction in either the entire sample or subset restricted to fear‐based diagnoses only. Furthermore, we hypothesized that individual differences in the continuation of treatment gains during the follow‐up period may be associated with genetic variation in ECB genes. Six independent clumps were nominally associated (*P *< 0.05) with change in symptom severity over the follow‐up period in the entire sample, five where an increasing number of copies of the minor allele was associated with a poorer response (sentinel SNPs: rs806365; rs2501431; rs2070956; rs7769940; rs2209172), and one with a more favorable response (sentinel SNP: rs6928813). Again, none of these effects survived multiple testing corrections. Three of these same sentinel SNPs were also nominally associated with response in the fear‐based subset (rs806365; rs7769940; rs2501431). The effect size of these SNPs was larger in the fear‐based subset with the effect of rs806365 remaining significant after multiple testing corrections were applied. Gene based tests of association were all non‐significant. In summary, our findings suggest only very limited evidence for a role of genetic variation in the ECB system in predicting individual differences in response to CBT for anxiety disorders in children and adolescents. Where these effects do exist they are very small and appear to have greater predictive power when examined in a sample restricted to fear‐based anxiety diagnoses only.

The strongest finding in our analyses was for SNP rs806365, which was nominally associated with a poorer response during the follow‐up period in the full sample (*P *= 0.004) and remained significantly associated after multiple testing correction in the fear‐based anxiety diagnosis subset (*P *= 0.0011). While not previously investigated with respect to anxiety linked traits or fear extinction, this locus has shown preliminary evidence of association with insulin resistance, risk for Type 2 diabetes and coronary heart disease [de Miguel‐Yanes et al., 2011]. Of greater relevance is research suggesting that variation at this locus may be associated with differential response to smoking cessation treatments and thus it could be hypothesized, sensitivity to environmental influences such as treatment regimens. For example, male carriers of one or more minor T alleles had increased rates of abstinence to treatment with buproprion (a norepinephrine and dopamine reuptake inhibitor) and transdermal (patch) nicotine replacement therapy but significantly decreased odds of abstinence in response to nicotine nasal spray replacement therapy [Lee et al., 2012]. In the present study, with each additional T allele, participants showed a significantly poorer response (a smaller reduction in severity) to treatment across the follow‐up period. This may indicate that T allele carriers are less sensitive to any continuing effects of CBT beyond the initial treatment period and ultimately may be placed at a greater risk of relapse. One possible mechanism worthy of further investigation is that this SNP (and the ECB system more broadly) may be involved in the maintenance of extinction memories beyond the active treatment period. While not possible in this study, it would be of interest to observe whether T allele carriers are at an increased risk of relapse with a longer follow‐up assessment period perhaps as a consequence of increased risk for spontaneous recovery of conditioned fear responding.

None of the SNPs previously studied in candidate gene studies of laboratory based fear extinction [Heitland et al., 2012; Dincheva et al., 2015], emotional processing [Chakrabarti et al., 2006; Domschke et al., 2008; Hariri et al., 2009; Gunduz‐Cinar et al., 2013] or response to antidepressant treatment [Domschke et al., 2008; Mitjans et al., 2012, 2013], on which data was available in this study, approached significance in either analysis. The only exception was rs2501431, a SNP in CNR2 that was previously studied in relation to response to treatment with citalopram in a small sample of outpatients with depression [Mitjans et al., 2012]. In this earlier study, variation in rs2501431 was not associated with symptom change in response to citalopram, but overall AA homozygotes reported more severe depression across the entire treatment period. In the present study, GG homozygotes showed a less favorable response (a smaller reduction in severity) during the follow‐up period, albeit only at a nominal level of significance. Unlike prior research, there was no significant difference in severity of anxiety at baseline or mean severity across the treatment and follow‐up period as a function of rs2501431 genotype. Differences in phenotype, sample type, treatment approach and sample size may explain the inconsistency in direction of effects seen across this study and that of Mitjans et al. [2012].

There are several explanations, which may in part account for the lack of convincing significant findings in this study despite encouraging experimental work for a role of genetic variation in ECB genes in fear extinction and emotional processing. Firstly, the CBT protocols given to participants, while strongly underpinned by the principles of extinction through exposure, also comprised a number of cognitive elements including teaching of coping skills and cognitive restructuring. Inevitably, this creates a far noisier analogue of the fear extinction paradigms used in the laboratory environment, which may have reduced the ability to detect significant effects. Furthermore, previous associations between variation in CNR1 and FAAH and fear extinction have been observed in response to short‐term experimentally conditioned fears in adults and not to clinical levels of anxiety in children and adolescents. Nonetheless, stronger effects may have been observed on response to a purer exposure‐based treatment or with a sample that was less heterogeneous with regard to anxiety diagnosis and treatment modality. In particular, the present sample and our initial analyses included the full range of anxiety disorder diagnoses. One possibility is that extinction learning may be implicated more or less in the mechanisms of treatments for different disorders. For example, extinction learning may be of greater relevance for the successful treatment of predominantly fear based disorders such as specific phobias and to a lesser extent for distress based disorders like generalised anxiety disorder [Borkovec and Ruscio, 2001]. A secondary analysis performed in the subset of the sample restricted to those with a fear‐based anxiety diagnosis provides suggestive evidence that this may be the case. The magnitude of effects was somewhat stronger in this restricted sample with the effect of rs806365 remaining significant even after multiple testing corrections were applied (see Table III). Further research should also establish a role for the ECB system in fear extinction in children and adolescents given that all of the experimental and treatment research to date has been with adult samples.

Secondly, any effects of ECB genetic variation on the maintenance of treatment gains, or conversely relapse of symptoms, may require a longer follow‐up period to emerge (90% of the current sample had a follow‐up period of 6 months or less). The change in symptom severity over the follow‐up period was smaller and less variable than that seen during the active treatment phase with 47% of participants showing no change in symptom severity from post‐treatment to follow‐up. Only a minority of participants (17%) showed any worsening of symptoms over the follow‐up period. Thus, analyses of the follow‐up period were limited by the reduced variance in response.

A more general limitation of the present study is that it took a candidate gene approach. However, this limitation was mitigated by the inclusion of array data on a subset of the sample providing more comprehensive coverage of the genes under investigation and LD context for the calculation of multiple testing corrections. Nonetheless within psychiatric genetics broadly, and the therapygenetics literature to date, candidate gene studies have often failed to replicate, have typically reported very small effect sizes and are sensitive to publication bias [Duncan and Keller, 2011; Lester and Eley, 2013]. Nominating candidate genes for investigation requires knowledge of the pathophysiology of the phenotype under investigation and the putative mechanisms through which CBT may act. Psychological treatment response is a complex trait and while extinction learning is an important process underpinning CBT, the etiology of treatment response is multifactorial. Thus, it is very unlikely that any single genetic polymorphism within the ECB system, or more generally, will explain a sufficiently large amount of variance in response to be clinically meaningful. To date, the strongest evidence for a role of the ECB system in fear extinction has come from animal studies employing genetic deletion and pharmacological modulation designs [Lafenetre et al., 2007]. Such studies are more likely to show large and pervasive effects in comparison to human genetic association studies, where the biological effect of an individual variant in vivo is likely to be very small. Despite being by far the largest therapygenetics study to date, the present study was powered to detect a variant capturing 1.6% of variance in treatment response with 80% power but had only 1.5% power to detect a variant of very small effect size explaining 0.1% of variance. If the true effect of rs806365 lies closer to the effect size of 0.0029% observed (in the full sample), then this would require a sample of 5,435 to detect these effects at α = 0.0017 with 80% power. Notwithstanding the huge expense and effort that would be required to assemble samples of this magnitude, such a small effect on its own is extremely unlikely to be of any clinical utility.

Given the challenges of candidate gene studies, it will become increasingly important for the therapygenetics field to work collaboratively to assemble large datasets that can be used to both study the mechanisms underlying CBT response and which will allow us to exploit hypothesis‐free whole genome based approaches. These methods have the potential to identify novel and unexpected variants associated with treatment response [Coleman et al., 2016]. In conjunction with statistical approaches such as polygenic risk scoring, genome wide approaches allow the opportunity to move beyond single variant approaches to methods which aggregate across a large number of markers in order to capture a greater and ultimately clinically significant proportion of the variance in outcome [Krapohl et al., 2015; Keers et al., 2016]. An interesting avenue for further research is to investigate epigenetic and gene expression predictors and correlates of psychological treatment response, as these approaches may allow us to get closer to the biological mechanisms of CBT response. This work while in its infancy has shown early promise [Perroud et al., 2013; Roberts et al., 2015, 2014; Yehuda et al., 2015]. Of relevance, a recent study investigating gene expression change in response to exposure‐based CBT for anxiety disorders reported that an increase in DALGB gene expression (diacylglycerol lipase beta gene), which is involved in the biosynthesis of 2‐AG (an endogenous endocannabinoid), was associated with greater reductions in severity while a reduction in DALGB expression corresponded with lower reductions in severity [Roberts et al., 2016]. This finding is consistent with research showing that increased levels of 2‐AG (an endogenous endocannabinoid) are associated with anxiolytic effects [Gunduz‐Cinar et al., 2013].

In summary, this is the first study to investigate the role of genetic variation in the endocannabinoid system and response to psychological therapy for anxiety disorders. A small number of genetic variants were nominally associated with individual differences in treatment response during the active treatment and follow‐up period. Only one of these effects remained significant after multiple testing corrections and in a sample restricted to those with a fear‐based anxiety disorder diagnosis. The ECB system remains a plausible target for involvement in response to psychological therapies underpinned by the principles of extinction learning. However, the effect of any single variant is likely to be very small given the complexity and multitude of mechanisms underpinning response to psychological treatments. The use of larger samples with greater statistical power and more homogeneous samples which reduce noise in the data would allow us to estimate the effect size of any variant with greater precision. Notwithstanding this, there are potentially large benefits for patients and wider society in knowing more about what determines who responds well to psychological therapies, and why. Thus, therapygenetics remains an important area for further research.

## Supporting information

Additional supporting information may be found in the online version of this article.

Supporting Information.

## Notes

1 In cases with primary selective mutism, a diagnosis of severe social phobia was also given. The selective mutism was considered by the clinician to be primary, the most interfering.
