# Supplementary material for: Genetic variation in the endocannabinoid system and response to Cognitive Behavior Therapy for child anxiety disorders
Source: Am J Med Genet B Neuropsychiatr Genet. 2016 Jun 27;174(2):144–55. doi: 10.1002/ajmg.b.32467 (PMC5324578; doi:10.1002/ajmg.b.32467)
Supplement: Supplementary file 1 — Supporting Information. [file AJMG-174-144-s001.docx]

**Supplementary Information**

**Methods and Materials**

Unless otherwise specified, clinical trials included all primary anxiety disorder diagnoses. All sites made secondary anxiety disorder diagnoses where appropriate.

**Sydney, Australia** (n = 641)

Participants aged 6-18 were recruited from the Centre for Emotional Health, Macquarie University, Sydney. All participants completed the Cool Kids program [Rapee et al 2006a], with 10-12 family sessions involving the parents (the majority of which were conducted in groups; 8% of the sample’s DNA were collected retrospectively). Variations on this treatment program include a subgroup from previous randomised trials who received group, individual or phone-based CBT sessions [Rapee et al 2006b]; participants from a guided self-help trial with phone support for children in rural Australia [Lyneham et al 2006]; a group from a trial with additional parental anxiety management [Hudson et al 2013]; and those recruited from an ongoing randomised trial of progressive allocation to treatment (stepped care).

**Reading and Oxford, UK** (n = 302 & 15)

Participants aged 5-18 were recruited jointly from Reading and Oxford from eight trials at the Berkshire Child Anxiety Clinic (University of Reading) and the Oxfordshire Primary Child and Adolescent Mental Health Service. Participants received treatment in three main themes; one focusing on children with anxious mothers; a set of trials using a parent-guided self-help CBT program; and an online CBT program for adolescents.

*The Mother and Child (MaCh) project* [Creswell et al 2015] Children whose mother also had a current anxiety disorder completed an 8 session manual-based CBT treatment based on the Cool Kids program. The mothers of these children also received extra sessions focussing on their own anxiety and on mother-child interactions.

*Overcoming.* Children were treated with a parent-guided self-help CBT program, comprised of the same primary components as the Cool Kids program [Thirlwall et al 2013]. This consisted of 2-4 in-person sessions and 2-4 telephone sessions. A subset of this group with a primary anxiety disorder diagnosis of Social Phobia also received targeted Cognitive Bias Modification Training (CBM-I, [Orchard et al In Submission]; Vassilopoulos et al [2009]. Additionally, participants with highly anxious parents (screened using DASS or by meeting ADIS criteria) were randomised to groups in a trial including additional sessions for the parents, which focused on strategies for tolerating children’s negative emotions [Hiller et al In Submission]. In Oxford, treatment was based on the same basic program, and delivered by primary health workers as part of a feasibility trial [Creswell et al 2010].

*BRAVE*. The final treatment group completed a therapist-supported online CBT program for adolescents (BRAVE, Spence et al [2011]), consisting of 10 sessions, half with 5 additional parent sessions and half without parent sessions.

**Aarhus, Denmark** (n = 123)

Participants aged 7-17 years were recruited from the Department of Psychology and Behavioural Sciences, Aarhus University, and all anxiety disorder diagnoses were included. Participants received CBT using the Cool Kids manual (including the adolescent version where appropriate [Rapee et al 2006c]). Participants came from two groups; one aged 7-17, from a trial including treatment and waitlist conditions; and another group aged 7-12 from a trial comparing efficacy of traditional group-based treatment with Cool Kids versus a guided self-help version with clinician support (bibliotherapy). In both trials only participants that received in-person CBT were included.

**Bergen, Norway** (n = 39)

Participants aged 5-13 were recruited from the child part of the “Assessment and Treatment – Anxiety in Children and Adults” study, Haukeland University Hospital, Bergen. Patients referred to outpatient mental health clinics in Western Norway, with a primary diagnosis of separation anxiety, social phobia, or generalized anxiety, received group or individual treatment with the FRIENDS program (4^th^ edition [Barrett 2004; Barrett et al 2006]) in a randomised control trial comparing active treatment with a waitlist condition [Wergeland et al 2014].

**Bochum, Germany** (n = 52)

Participants aged 5-18 were recruited from the Research and Treatment Centre for Mental Health, Ruhr-Universität Bochum. Participants received either exposure-based CBT (8-25 sessions, with sessions occurring at least every 2 weeks), the Coping Cat program [Kendall 1994], or a family-based version of CBT specifically designed to target separation anxiety disorder (TAFF [Schneider et al 2013a; Schneider et al 2013b]). Diagnoses were provided separately for parent- and child-report. The primary diagnosis was selected as being the most severe from either reporter. If the most severe disorder reported by each was of equal severity but was a different diagnosis, the parent-reported diagnosis was selected.

**Groningen, the Netherlands** (n = 36)

Participants aged 8 to 17 were recruited from the Department of Child and Adolescent Psychiatry, University of Groningen. All participants were treated within a randomised control trial of Coping Cat (Dutch version [Nauta et al 1998] including 12 individual child sessions and 2 parent sessions.

**Florida, USA** (n = 38)

Participants aged 7 to 16 (including all primary anxiety disorder diagnoses except PTSD) were recruited from the Child Anxiety and Phobia Program, Florida International University, Miami. All participants received 12 to 14 hour-long sessions of individual manualised CBT. Additionally, two conditions included parental involvement focussing on different parent skills (Relationship Skills Training or Reinforcement Skills Training).

**Basel, Switzerland (n = 47)**

Participants aged 5-13 (all with a primary diagnosis of Separation Anxiety Disorder) were recruited from the Faculty of Psychology, University of Basel. All participants took part in a randomised control trial comparing a family-based version of CBT specifically designed to target separation anxiety disorder (TAFF[Schneider et al 2013a; Schneider et al 2013b]) with Coping Cat [Kendall 1994]. All participants received 16 sessions over 12 weeks.

**Cambridge, UK (n = 12)**

Participants aged 8–17 were recruited from the Medical Research Council Cognition and Brain Sciences Unit, Cambridge, UK. Participants were taking part in the Acute Stress Programme for Children and Teenagers (ASPECTS) trial, which recruited individuals exposed to a recent (i.e. in the previous six months) traumatic stressor (i.e. any event that involve the threat of death, severe injury, or threat to bodily integrity, or witnessing such an event). Those that developed PTSD were randomized to a 10-week waitlist or individual PTSD-specific CBT,[Smith et al 2007] which consisted of up to 10 sessions over a 10-week period. Only participants that received treatment were included.

**Amsterdam, the Netherlands (n = 4)**

Participants aged 10–14 were recruited from the Academic Treatment Centre for Parent and Child, University of Amsterdam (UvA) Minds and received either 12 weeks of CBT in individual sessions or 8 weeks of CBT in group sessions, according to the Dutch protocol “Discussing + Doing = Daring.”[Bögels 2008] Treatment was coded as low parental involvement. Diagnoses were provided separately for parent and child report, with the primary diagnosis selected from these data by the trial manager.

**Supplementary Results**

**Change in symptom severity from baseline to post-treatment and post-treatment to follow-up: analyses restricted to White European ancestry subset (N = 916)**

When the analyses were restricted to a subset that identified as having four White European grandparents (n = 916), none of the SNPs genotyped in the entire sample were significantly associated with treatment response across the active treatment period(all *p* values > .05). However rs2501431 (*p* = 0.07) was situated in an independent clump that was nominally associated with a more favourable treatment response (sentinel SNP rs35385477, see Table S2 for clump based test statistics). For the post-treatment to follow-up time period, three independent clumps were nominally associated with a poorer response (sentinel SNPs: rs806365; rs2501431; rs2070953), while one independent clump predicted a more favourable response (sentinel SNP: rs2023239, see Table S1). The top SNPs identified in the White European subset corresponded to the 1^st^, 2^nd^, 3^rd^ and 4^th^ clumps identified in the entire dataset.

Table SI: Sample characteristics by site for the subset of the sample with a fear-based anxiety disorder diagnosis

| **Characteristic** | **Sydney** | **Reading** | **Aarhus** | **Bergen** | **Bochum** | **Basel** | **Groningen** | **Oxford** | **Florida** | **Amsterdam** | **Total** |
| --- | --- | --- | --- | --- | --- | --- | --- | --- | --- | --- | --- |
| N | **265** | **202** | **83** | **29** | **47** | **47** | **29** | **13** | **30** | **4** | **749** |
| Gender  Female n (%) | 129 (48.7) | 109  (54.0) | 46 (55.4) | 19 (65.5) | 27 (57.4) | 26 (55.3) | 16 (55.2) | 7 (60.0) | 16 (53.8) | 0 (0) | 395 (52.7) |
| Age: mean (SD) | 9.30 (1.86) | 9.59 (1.71) | 10.99 (2.40) | 11.38 (1.99) | 10.91 (2.56) | 8.49 (2.07) | 11.69 (3.08) | 8.77 (1.48) | 9.40 (2.19) | 12.00 (1.83) | 9.80 (2.19) |
| Severity primary diagnosis: mean (SD) | 6.36 (0.88) | 5.65 (0.83) | 6.67 (1.24) | 6.52 (1.30) | 6.77 (1.15) | 5.98 (0.77) | 6.28  (0.96) | 5.54 (0.97) | 6.97 (1.16) | 5.75 (1.71) | 6.21 (1.05) |
| Primary diagnosis: n (%) | |  |  |  |  |  |  |  |  |  |  |
| SoAD | 136 (51.3) | 62 (30.7) | 18 (21.7) | 17 (58.6) | 15 (31.9) | 0 (0) | 14 (48.3) | 6 (46.2) | 10 (33.3) | 1 (25.0) | 279 (37.2) |
| SP | 51 (19.2) | 49 (24.3) | 19 (22.9) | 0 (0) | 17 (36.2) | 0 (0) | 6 (20.7) | 1  (7.7) | 5  (16.7) | 1  (25.0) | 149 (19.9) |
| SAD | 74  (27.9) | 77  (38.1) | 37  (44.6) | 12  (41.4) | 13  (27.7) | 47  (100) | 6  (20.7) | 6  (46.2) | 10  (33.3) | 2  (50.0) | 284 (37.9) |
| Other AD^a^ | 4  (1.5) | 14  (6.9) | 9  (10.8) | 0  (0) | 2  (4.3) | 0  (0) | 3  (10.3) | 0  (0) | 5  (16.7) | 0  (0) | 37  (4.9) |
| CBT treatment: n (%) | |  |  |  |  |  |  |  |  |  |  |
| Individual based | 9  (3.4) | 86  (42.6) | 2  (2.4) | 17  (58.6) | 47  (100) | 47  (100) | 29  (100) | 0  (0) | 30  (100) | 1  (25.0) | 268 (35.8) |
| Group-based | 224  (84.5) | 0  (0) | 81  (97.6) | 12  (41.4) | 0  (0) | 0  (0) | 0  (0) | 0  (0) | 0  (0) | 3  (75.0) | 320 (42.7) |
| Guided self-help | 32  (12.1) | 116  (57.4) | 0  (0) | 0  (0) | 0  (0) | 0  (0) | 0  (0) | 13  (100) | 0  (0) | 0  (0) | 161 (21.5) |

Other anxiety disorders include Panic Disorder and Selective Mutism.

Table SII: Independent clumps nominally associated (*p* < .05) with treatment response between a.) baseline and post-treatment and b.) post-treatment and follow-up in the White European ancestry subset

|  | a.) **Change in symptom severity from baseline to post-treatment** | | | | | | | |  |  |
| --- | --- | --- | --- | --- | --- | --- | --- | --- | --- | --- |
| **Sentinel SNP** | | **Gene** | **Clump BP** | **Minor allele** | **MAF** | | **Info** | **β** | **95% CI** | ***p*** |
| rs35385477 | | CNR2 | 24108683-24206032 | G | 0.454 | | 0.989 | -0.05 | -0.10 - -0.01 | .030^a^ |
|  | b.) **Change in symptom severity from post-treatment to follow-up** | | | | | | | |  |  |
| rs806365 | | CNR1 | 88843390-88845949 | T | | 0.395 | Genotyped (microarray) | 0.13 | 0.04 – 0.22 | .004 |
| rs2501431 | | CNR2 | 24108683-24206032 | G | | 0.435 | Genotyped (LGC) | 0.11 | 0.03 –0.19 | .009 |
| rs2070953 | | CNR2 | 24191219-24223859 | C | | 0.103 | 0.995 | 0.18 | 0.04 – 0.32 | .010 |
| rs2023239 | | CNR1 | 88861208-88885326 | C | | 0.183 | 0.966 | -0.12 | -0.23 − -0.00 | .047 |

Note: All genotypes were coded to reflect an additive model where -1 = common homozygote, 0 = heterozygote and 1 = rare homozygote

Regression weights (**β**) significantly less than 0 indicate that the minor allele of that SNP was associated with a greater reduction in symptom severity across the active treatment or follow-up period. Values significantly greater than 0 indicate that the minor allele of that SNP was associated with a poorer reduction in symptom severity.

^a^ This clump contains the directly genotyped SNP rs2501431. Test statistics for this SNP are β = -0.04, 95% CI: -0.08 *–* 0.004, *p* = .073.

Table SIII: Statistics for all clumps (identified by sentinel SNP) with treatment response between baseline and post-treatment for the full sample

| **Sentinel SNP (Gene)** | **Clump BP** | **MAF/Minor allele** | **β** | **95% CI** | ***p*** | **n^a^** | **Additional SNPs included in clump**  **(SNPs with *p* values < .05 highlighted in bold, total number of additional SNPs in clump given in brackets e.g. (74))** |
| --- | --- | --- | --- | --- | --- | --- | --- |
|  |  |  |  |  |  |  |  |
| rs12133557  (CNR2) | 24191219 - 24223859 | 0.098  T | -0.07 | -0.14– -0.01 | 0.020 | 925 | **rs74937660**, rs74223776, rs3003325,rs3003328, rs3003329, rs3003621, rs3003332, rs2070956, rs2070955, rs2070954, rs2070953, rs3003320, rs3003622, rs3003321, rs6664030, rs2503003, **exm31621**, rs12754324, rs12727867, rs28404091, rs7541711, rs7541713, rs7541819, rs7532916, rs7541841, rs12741866, rs12759455, rs12733278, rs12759917, rs4625225, rs60390132, rs7519729, rs111834151, rs12742876, rs61778195, rs5026902, rs4648919, rs143281762, rs2501398, rs6424127, rs6424128, rs6424129, rs6424130, rs7512349, rs2502967, rs2501399, rs2501400, rs2502968, rs2501401, rs2502969, rs2502970, rs2501402, rs6424131, rs7415219, rs7550908, rs7550371, rs7537224, rs12730734, rs201557597, rs2501403, rs3003326, rs28735813, rs35829803, rs9424397, rs9424398, rs12724034, kgp1774784, rs2502971, rs2502972, rs9424400, rs9424338, rs2501404, rs3003327, rs3003623, rs2502973, rs2502974 (76) |
| rs6454676  (CNR1) | 88860482- 88885426 | 0.104  A | 0.07 | 0.002 – 0.13 | 0.042 | 926 | rs2023239, **rs1535255**, rs6928499, rs6928813, rs6912668, rs9450901, rs9450902, rs9444586, rs9450903, rs9450904, rs10485170, rs74471317, rs9450906, rs9444587, rs9450907, rs11966501, rs11968764, rs11963892, rs78335089, rs9444588, rs6454678 (21) |
| rs45540335  (FAAH) | 46766751 - 46865387 | 0.121  A | -0.05 | -0.11-0.00 | 0.066 | 927 | rs3753362, rs79783387, exm56515, rs77526109, rs76976909, rs76771627, exm56596, rs41294484, rs59082884, rs4110477, rs3891758, rs45480993, rs324416 (13) |
| rs12197767  (CNR1) | 88907388 - 88941764 | 0.245  T | 0.03 | -0.01-0.07 | 0.173 | 924 | rs9450916, rs16880378, rs2038447, rs12212677, rs7766691, rs7752742, rs9362470, rs1555340, rs9294398, rs1358791, rs1324958, rs1324957, rs9344763, rs9344764, rs9353531, rs9362472, rs9344765, rs9351142, rs9351143, rs9344767 (20) |
| rs58370001  (CNR1) | 88919215 - 88940323 | 0.067  C | -0.05 | -0.13 –0.02- | 0.177 | 924 | rs7754491, rs7769918, rs56396859, rs9450925, rs59449423, rs55882449, rs60315037, rs113006189, rs1216678, rs16880396, rs145705030, rs7756920, rs7757556, rs62431489, rs6927294, rs62431491, rs964647, rs59903039, rs6906154 (19) |
| exm56547  (FAAH) | 46806959 - 46882753 | 0.329  G | 0.03 | -0.01-0.07 | 0.186 | 939 | rs11579255, rs34160166, rs2145409, rs11802866, rs12404971, rs12040179, rs10252, rs12075550, rs141064494, rs10890390, rs10890391, rs11211270, rs11804189, rs56733721, rs3863641, rs12073998, rs11211271, rs4660925, rs35056299, rs913168, rs11589812, rs4141964, rs3766246, rs2295633, rs11576941, rs61784641, rs55646923 (27) |
| rs4649123  (CNR2) | 24108683 - 24206032 | 0.432  A | -0.02 | -0.06 –0.02 | 0.237 | 929 | rs10917425, rs12031592, rs2256179, rs974698, rs1018396, rs2502986, rs6679378, rs2502987, rs2982390, rs2473377, rs2179395, rs2502979, rs2502980, rs34883557, rs6697805, rs6424115, rs60013677, rs34477640, rs7519554, rs35385477, rs6424116, rs71575777, rs10917430, exm31570, rs12748109, rs11803575, rs12755062, rs12141409, rs34570472, rs10799804, rs3123554, rs4483353, rs2503002, rs2503001, rs2503000, rs2502999, rs2502998, rs2502997, rs2501417, rs6680132, rs6672157, rs2501423, rs2502996, rs2502995, rs2501425, rs6663474, rs3003334, rs35955796, rs3003335, rs6665733, rs1130321, rs1130320, rs1106, rs1105, rs2229585, rs2229584, rs2229583, rs2229581, rs2229580, rs2502993, rs4649124, rs3003336, rs2501431, rs2502992, exm31668, rs2502991, rs6667839, rs6672499, rs2501433, rs6658703, rs2501434, rs6673210, rs3123555, rs3123556, rs6424119, rs6424120, rs4341315, rs2501367, rs2502990, rs2502989, rs2502988, rs2501369 (82) |
| rs1886117  (FAAH) | 46954587 - 46974497 | 0.315  T | 0.02 | -0.02-0.06 | 0.264 | 929 | rs1998545, rs7531088 (2) |
| rs6676038  (FAAH) | 46761389 - 46798466 | 0.017  A | -0.08 | -0.23-0.06 | 0.276 | 933 | rs7548226, rs144930516, rs142618742, rs7542864, rs7545120, rs200232349, rs17102133, rs114531159, rs140815810, rs199585121, rs112746922, rs112387082, rs147637102 (13) |
| rs55845894  (CNR1) | 88947649 - 88971424 | 0.146  C | 0.03 | -0.03 –0.08 | 0.294 | 920 | rs11966650, rs7769940, rs28816226, rs6910128, rs2325103, rs7753442, rs35750466, rs6922315, rs5878063, rs3929734, rs34367043, rs59413611, rs6918613, rs7765818, rs61310563, rs76763854, rs7762344, rs59838907, rs1408701, rs7453339, rs6454683, rs6454685 (22) |
| rs806379  (CNR1) | 88858648 - 88867925 | 0.449  T | 0.02 | -0.02-0.05 | 0.295 | 1022 | rs806376, rs12205430 (2) |
| rs806374  (CNR1) | 88850150 - 88857320 | 0.346  C | -0.02 | -0.06 –0.02 | 0.331 | 939 | rs806368 (1) |
| rs2180619  (CNR1) | 88877952 | 0.400  G | 0.01 | -0.02-0.05 | 0.429 | 1162 | NONE |
| rs1408702  (CNR1) | 88973751 | 0.442  G | 0.01 | -0.02-0.05 | 0.45 | 939 | NONE |
| rs6683116  (FAAH) | 46975773 - 46975877 | 0.189  T | 0.02 | -0.03-0.07 | 0.455 | 939 | rs6698196 (1) |
| exm2268681  (FAAH) | 46938837 – 46978946 | 0.375  C | 0.01 | -0.02 –0.05 | 0.457 | 939 | rs2031247, rs199790074, rs12132747, rs4660353, rs2209172, rs56393814 (6) |
| rs10890388  (FAAH) | 46761496 - 46888039 | 0.240  T | -0.02 | -0.06-0.03 | 0.459 | 921 | rs6678149, rs2145408, rs6429600, exm56619, rs324420, rs324418, rs12029329, rs201392030, rs4660928, rs6670926, rs4660346 (11) |
| rs6908693  (CNR1) | 88817588 - 88817934 | 0.127  A | -0.02 | -0.08 –0.04 | 0.478 | 931 | rs6908755, rs6913146 (2) |
| rs9344758  (CNR1) | 88894422 - 88903557 | 0.462  T | -0.03 | -0.11-0.05 | 0.492 | 938 | rs1535257, rs7747006, rs7751075, rs16880345, rs2038448, rs9294397, rs2875545, rs57809420, rs12209554, rs9353529, rs6920617, rs35670824, rs719537, rs1321361, rs9351140, rs9362468, rs9450914, rs9450915, rs2325098, rs2325099, rs12213790, rs2325100 (22) |
| rs324410  (FAAH) | 46834173 - 46888905 | 0.056  T | -0.01 | -0.05-0.02 | 0.492 | 925 | rs324425, rs324424, rs324423, rs324421 (4) |
| rs9450877  (CNR1) | 88796053 - 88797885 | 0.334  A | -0.01 | -0.05 –0.03 | 0.505 | 938 | rs9450876, rs1324075 (2) |
| rs806365  (CNR1) | 88843390 - 88845949 | 0.405  T | -0.01 | -0.05-0.03 | 0.509 | 939 | rs10485171 (1) |
| rs806371  (CNR1) | 88856363 | 0.110  G | 0.01 | -0.04 –0.07 | 0.684 | 974 | NONE |
| rs4707441  (CNR1) | 88899850 - 88904498 | 0.152  G | 0.01 | -0.04-0.06 | 0.695 | 939 | rs9450913, rs4707442 (2) |
| rs2281774  (FAAH) | 46905802 - 46928349 | 0.214  T | 0.01 | -0.04-0.06 | 0.711 | 920 | rs11211276, rs12407178, rs12409747, rs7538292, rs3795315, rs72637962, rs12126376, rs11211278, rs11293072, rs2281775, rs4660347, rs942258, rs10890397, rs4660933, rs2031248, rs4660348, rs11211282 (17) |
| rs56953705  (FAAH) | 46937668 - 46960043 | 0.016  T | -0.02 | -0.17-0.12 | 0.749 | 923 | rs74810435, rs76181455, rs60712134, rs112699995, rs74342198 (5) |
| rs55939860  (FAAH) | 46806242 - 46886953 | 0.278  G | -0.01 | -0.05-0.03 | 0.775 | 923 | rs67545510, rs112712935, rs41293273, rs41293275, rs17361749, rs41293277, rs10489769, rs72885163, rs68083747, rs66972124, rs17357621, rs56909107, rs41293285, rs17357628, rs17357635, rs68191463, rs41293287, rs56126529, rs7525309, rs7548675, rs7515284, rs41294456, rs66516678, rs41294458, rs79697925, rs17361763, rs6684274, rs11304172, rs6696777, rs6659228, rs10661193, rs6697123, rs67487250, rs113610895, rs112280595, rs112491752, rs112089006, rs72677585, rs17357676, rs17361791, rs41294460, rs72885198, rs79490411, rs7556425, rs61358317, rs72886903, rs68112720, rs17361805, rs72677587, rs17361812, rs66575205, rs72886907, rs111226885, rs111699884, rs72886911, rs56344958, rs56121132, rs5013329, rs5013330, rs17357683, rs6667861, rs1057533, rs1057534, rs1057535, rs1135459, rs1135460, rs1475390, rs5773907, rs1475389, rs1475388, rs57267378, rs58162700, rs1812705, rs966907, rs112324418, rs72677588, rs72677589, rs66828173, rs72677590, rs72677591, rs72677592, rs41294476, rs41294478, rs17413701, rs7515598, rs7529674, rs12385696, rs12385697, rs12385693, rs12385695, rs28699008, rs56179746, rs146398931, rs6679080, rs6690075, rs6666601, rs6682266, rs6679898, rs10158236, rs10157084, rs112980938, rs56363851, rs66990604, rs201127808, rs10158572, rs10158130, rs10157464, rs56030283, rs55971480, rs66911505, rs6659448, rs6677394, rs6674726, rs112911951, rs113033705, rs72677593, rs72677594, rs7520482, rs7520497, rs7532149, rs28578741, rs202085913, rs55999016, rs56063031, rs67276232, rs6669025, rs200601367, rs55887761, rs9661240, rs1053624, rs1053627, rs1053628, rs6429599, rs17361819, rs67142569, rs17361833, rs41534051, rs17357711, rs6683192, rs12062, rs111725921, rs66847432, rs67547686, rs113076306, rs17357759, rs17361873, rs72677596, rs72677599, rs72677600, rs17361887, rs55670684, rs56049453, rs67200518, rs72677602, rs67941619, rs66483119, rs55668511, rs55693298, rs68012736, rs143856001, rs144535648, rs147142636, rs66473412, rs6659681, rs6659788, rs55921163, rs55926300, rs111868160, rs13374893, rs13374968, rs6694628, rs6658556, rs6695043, rs72679807, rs56284503, rs17361915, rs113623605, rs144250006, rs113534835, rs56358525, rs6681857, rs56349187, rs79610407, rs72890715, rs4372193, rs111723629, rs3991877, rs57240150, rs113887859, rs201070992, rs61519400, rs56306849, rs1984491, rs1984490, rs45449893, rs45517837, rs932816, rs72890727, rs6674305, rs56130131, rs11288511, rs6703374, rs6703669, rs45524035, rs17361936, rs17361950, rs6662982 (207) |
| rs3766248  (FAAH) | 46773488 | 0.021  A | 0.01 | -0.11 –0.14 | 0.831 | 935 | NONE |
| rs1049353  (CNR1) | 88853635 | 0.274  A | 0.00 | -0.04-0.04 | 0.959 | 1175 | NONE |
| rs10890398  (FAAH) | 46925594 | 0.180  C | 0.00 | -0.05-0.05 | 0.981 | 939 | NONE |

Note: All genotypes were coded to reflect an additive model where -1 = common homozygote, 0 = heterozygote and 1 = rare homozygote

Regression weights (***β***) significantly less than 0 indicate that an increasing number of copies of the minor allele of the SNP was associated with a greater reduction in symptom severity across the active treatment or follow-up period. Values significantly greater than 0 indicate that an increasing number of copies of the minor allele of the SNP was associated with a poorer reduction in symptom severity.

^a^ n reflects total number of cases included in regression analysis for the sentinel SNP.

Table SIV: Statistics for all clumps (identified by sentinel SNP) with treatment response between baseline and post-treatment in the subset of the sample with fear-based anxiety disorder diagnoses

| **Sentinel SNP (Gene)** | **Clump BP** | **MAF/Minor allele** | **β** | **95% CI** | ***p*** | **n^a^** | **Additional SNPs included in clump**  **(SNPs with *p* values < .05 highlighted in bold, total number of additional SNPs in clump given in brackets e.g. (74))** |
| --- | --- | --- | --- | --- | --- | --- | --- |
|  |  |  |  |  |  |  |  |
| rs12133557  (CNR2) | 24191219 - 24223859 | 0.094  T | -0.11 | -0.20 – -0.03 | .011 | 540 | **rs74937660**, **rs74223776**, **rs3003325**, **rs3003328**, **rs3003329**, **rs3003621**, **rs3003332**, **rs2070956**, **rs2070955**, **rs2070954**, **rs2070953**, **rs3003320**, **rs3003622**, **rs3003321**, **rs6664030**, **rs2503003**, **exm31621**, rs12754324, rs12727867, rs28404091, rs7541711, rs7541713, rs7541819, rs7532916, rs7541841, rs12741866, rs12759455, rs12733278, rs12759917, rs4625225, rs60390132, rs7519729, rs111834151, rs12742876, rs61778195, rs5026902, rs4648919, rs143281762, rs2501398, rs6424127, rs6424128, rs6424129, rs6424130, rs7512349, rs2502967, rs2501399, rs2501400, rs2502968, rs2501401, rs2502969, rs2502970, rs2501402, rs6424131, rs7415219, rs7550908, rs7550371, rs7537224, rs12730734, rs201557597, rs2501403, rs3003326, rs28735813, rs35829803, rs9424397, rs9424398, rs12724034, kgp1774784, rs2502971, rs2502972, rs9424400, rs9424338, rs2501404, rs3003327, rs3003623, rs2502973, rs2502974 (76) |
| rs6454676  (CNR1) | 88860482 - 88885426 | 0.108  A | 0.09 | 0.005 – 0.17 | .038 | 539 | rs2023239, rs1535255, rs6928499, rs6928813, rs6912668, rs9450901, rs9450902, rs9444586, rs9450903, rs9450904, rs10485170, rs74471317, rs9450906, rs9444587, rs9450907, rs11966501, rs11968764, rs11963892, rs78335089, rs9444588, rs6454678 (21) |
| rs6927294  (CNR1) | 88919215 - 88940323 | 0.068  T | -0.1 | -0.20-0.00 | .053 | 547 | rs7754491, rs7769918, rs56396859, rs58370001, rs9450925, rs59449423, rs55882449, rs60315037, rs113006189, rs1216678, rs16880396, rs145705030, rs7756920, rs7757556, rs62431489, rs62431491, rs964647, rs59903039, rs6906154 (19) |
| rs806379  (CNR1) | 88858648 - 88867925 | 0.435  T | 0.03 | -0.02-0.08 | .182 | 588 | rs806376, rs12205430 (2) |
| rs11966650  (CNR1) | 88947649 - 88971424 | 0.134  G | 0.05 | -0.03-0.13 | .218 | 538 | rs7769940, rs28816226, rs6910128, rs2325103, rs7753442, rs35750466, rs6922315, rs5878063, rs3929734, rs34367043, rs59413611, rs55845894, rs6918613, rs7765818, rs61310563, rs76763854, rs7762344, rs59838907, rs1408701, rs7453339, rs6454683, rs6454685 (22) |
| rs45540335  (FAAH) | 46766751 - 46865387 | 0.131  A | -0.05 | -0.12 - 0.03 | .219 | 542 | rs3753362, rs79783387, exm56515, rs77526109, rs76976909, rs76771627, exm56596, rs41294484, rs59082884, rs4110477, rs3891758, rs45480993, rs324416 (13) |
| rs16880345  (CNR1) | 88894422 - 88902091 | 0.201  G | -0.04 | -0.10 - 0.02 | .221 | 544 | rs1535257, rs7747006, rs7751075, rs9344758, rs2875545, rs12209554, rs9353529, rs6920617, rs35670824, rs719537, rs1321361, rs9351140, rs9362468, rs9450914, rs9450915, rs2325099 (16) |
| rs56733721  (FAAH) | 46806242 - 46888039 | 0.413  G | -0.03 | -0.08-0.02 | .222 | 543 | rs67545510, rs112712935, rs41293273, rs41293275, rs17361749, rs41293277, rs10489769, rs11579255, rs72885163, rs68083747, rs66972124, rs17357621, rs56909107, rs41293285, rs17357628, rs17357635, rs68191463, rs41293287, rs56126529, rs7525309, rs7548675, rs7515284, rs41294456, rs66516678, rs41294458, rs79697925, exm56547, rs17361763, rs6684274, rs11304172, rs6696777, rs6659228, rs10661193, rs6697123, rs67487250, rs113610895, rs112280595, rs112491752, rs112089006, rs72677585, rs17357676, rs17361791, rs41294460, rs72885198, rs79490411, rs7556425, rs61358317, rs72886903, rs68112720, rs17361805, rs72677587, rs17361812, rs66575205, rs72886907, rs111226885, rs111699884, rs72886911, rs56344958, rs56121132, rs5013329, rs5013330, rs17357683, rs6667861, rs1057533, rs1057534, rs1057535, rs1135459, rs1135460, rs1475390, rs5773907, rs1475389, rs1475388, rs57267378, rs58162700, rs1812705, rs966907, rs112324418, rs72677588, rs72677589, rs66828173, rs72677590, rs72677591, rs72677592, rs41294476, rs41294478, rs17413701, rs7515598, rs7529674, rs12385696, rs12385697, rs12385693, rs12385695, rs28699008, rs56179746, rs146398931, rs6679080, rs6690075, rs6666601, rs6682266, rs34160166, rs6679898, rs10158236, rs10157084, rs112980938, rs56363851, rs66990604, rs201127808, rs10158572, rs10158130, rs10157464, rs2145409, rs56030283, rs55971480, rs66911505, rs6659448, rs6677394, rs6674726, rs11802866, rs112911951, rs113033705, rs12404971, rs72677593, rs72677594, rs7520482, rs7520497, rs7532149, rs28578741, rs202085913, rs55999016, rs56063031, rs67276232, rs12040179, rs6669025, rs200601367, rs55887761, rs9661240, rs1053624, rs1053627, rs1053628, rs6429599, rs17361819, rs67142569, rs17361833, rs41534051, rs17357711, rs6683192, rs10252, rs12062, rs111725921, rs66847432, rs67547686, rs113076306, rs17357759, rs17361873, rs72677596, rs12075550, rs72677599, rs72677600, rs17361887, rs55670684, rs56049453, rs67200518, rs141064494, rs72677602, rs10890391, rs67941619, rs66483119, rs55668511, rs55693298, rs11211270, rs68012736, rs143856001, rs11804189, rs144535648, rs147142636, rs66473412, rs6659681, rs6659788, rs55921163, rs55926300, rs111868160, rs3863641, rs12073998, rs13374893, rs13374968, rs6694628, rs6658556, rs6695043, rs72679807, rs56284503, rs17361915, rs113623605, rs11211271, rs144250006, rs113534835, rs56358525, rs6681857, rs56349187, rs79610407, rs4660925, rs72890715, rs4372193, rs111723629, rs3991877, rs35056299, rs57240150, rs113887859, rs201070992, rs61519400, rs56306849, rs913168, rs1984491, rs1984490, rs11589812, rs45449893, rs45517837, rs932816, rs72890727, rs6674305, rs56130131, rs11288511, rs6703374, rs6703669, rs45524035, rs17361936, rs17361950, rs11576941, rs6662982, rs4660928, rs55939860, rs6670926, rs4660346 (232) |
| rs2502987  (CNR2) | 24108683 - 24206032 | 0.439  A | -0.03 | -0.08 - 0.02 | .262 | 537 | rs10917425, rs12031592, rs2256179, rs974698, rs1018396, rs2502986, rs6679378, rs2982390, rs2473377, rs2179395, rs2502979, rs2502980, rs34883557, rs6697805, rs6424115, rs60013677, rs34477640, rs7519554, rs35385477, rs6424116, rs71575777, rs10917430, exm31570, rs12748109, rs11803575, rs12755062, rs12141409, rs34570472, rs10799804, rs3123554, rs4483353, rs2503002, rs2503001, rs4649123, rs2503000, rs2502999, rs2502998, rs2502997, rs2501417, rs6680132, rs6672157, rs2501423, rs2502996, rs2502995, rs2501425, rs6663474, rs3003334, rs35955796, rs3003335, rs6665733, rs1130321, rs1130320, rs1106, rs1105, rs2229585, rs2229584, rs2229583, rs2229581, rs2229580, rs2502993, rs4649124, rs3003336, rs2501431, rs2502992, exm31668, rs2502991, rs6667839, rs6672499, rs2501433, rs6658703, rs2501434, rs6673210, rs3123555, rs3123556, rs6424119, rs6424120, rs4341315, rs2501367, rs2502990, rs2502989, rs2502988, rs2501369 (82) |
| rs806368  (CNR1) | 88850150 - 88857320 | 0.223  G | 0.03 | -0.02-0.08 | .276 | 695 | rs806371, rs806374 (2) |
| rs9450876  (CNR1) | 88796053 - 88797885 | 0.350  A | -0.03 | -0.08 - 0.02 | .285 | 545 | rs9450877, rs1324075 (2) |
| rs112699995  (FAAH) | 46937668 - 46960043 | 0.020  T | 0.1 | -0.08-0.28 | .297 | 540 | rs74810435, rs76181455, rs56953705, rs60712134, rs74342198 (5) |
| rs6683116  (FAAH) | 46975773 - 46975877 | 0.190  T | 0.03 | -0.03-0.10 | .300 | 547 | rs6698196 (1) |
| rs7548226  (FAAH) | 46761389 - 46798466 | 0.013  A | 0.11 | -0.11 - 0.34 | .334 | 544 | rs6676038, rs144930516, rs142618742, rs7542864, rs7545120, rs200232349, rs17102133, rs114531159, rs140815810, rs199585121, rs112746922, rs112387082, rs147637102 (13) |
| rs9450913  (CNR1) | 88899850 - 88904498 | 0.145  T | 0.03 | -0.04-0.11 | .342 | 538 | rs4707441, rs4707442 (2) |
| rs11211282  (FAAH) | 46905802 - 46928349 | 0.426  A | 0.02 | -0.03 - 0.07 | .37 | 547 | rs11211276, rs12407178, rs12409747, rs7538292, rs3795315, rs72637962, rs12126376, rs11211278, rs11293072, rs2281775, rs2281774, rs4660347, rs942258, rs10890397, rs4660933, rs2031248, rs10890398, rs4660348 (18) |
| exm2268681  (FAAH) | 46938837 – 46978946 | 0.389  C | 0.02 | -0.03 - 0.08 | .373 | 547 | rs2031247, rs199790074, rs12132747, rs4660353, rs2209172, rs56393814 (6) |
| exm56619  (FAAH) | 46761496 - 46882753 | 0.212  A | 0.03 | -0.04 - 0.09 | .400 | 547 | rs10890388, rs10890390, rs6678149, rs2145408, rs6429600, rs4141964, rs3766246, rs324420, rs324418, rs2295633, rs12029329, rs201392030, rs61784641, rs55646923 (14) |
| rs2180619  (CNR1) | 88877952 | 0.414  G | 0.02 | -0.03-0.06 | .470 | 675 | NONE |
| rs10485171  (CNR1) | 88843390 - 88845949 | 0.458  G | -0.01 | -0.06-0.04 | .601 | 547 | rs806365, rs1049353 (2) |
| rs12212677  (CNR1) | 88907388 - 88941764 | 0.463  G | -0.01 | -0.06-0.04 | .633 | 539 | rs9450916, rs16880378, rs2038447, rs7766691, rs7752742, rs9362470, rs9294398, rs1358791, rs1324958, rs1324957, rs9344763, rs9344764, rs9353531, rs9362472, rs9344765, rs9351142, rs12197767, rs9351143, rs9344767 (19) |
| rs1886117  (FAAH) | 46954587 - 46974497 | 0.306  T | 0.01 | -0.04-0.07 | .646 | 545 | rs1998545, rs7531088 (2) |
| rs324410  (FAAH) | 46834173 - 46888905 | 0.061  T | -0.02 | -0.13-0.09 | .68 | 539 | rs324425, rs324424, rs324423, rs324421 (4) |
| rs1408702  (CNR1) | 88973751 | 0.452  G | -0.01 | -0.06-0.04 | .726 | 547 | NONE |
| rs3766248  (FAAH) | 46773488 | 0.018  A | -0.03 | -0.21 - 0.15 | .751 | 544 | NONE |
| rs2038448  (CNR1) | 88898440 - 88902564 | 0.376  C | 0.01 | -0.04 - 0.06 | .785 | 538 | rs57809420, rs2325098, rs12213790 (3) |
| rs6908755  (CNR1) | 88817588 - 88817934 | 0.130  T | 0.01 | -0.07-0.09 | .808 | 547 | rs6908693, rs6913146 (2) |
| rs9294397  (CNR1) | 88898611 - 88903557 | 0.274  C | -0.01 | -0.06 - 0.05 | .829 | 547 | rs2325100 (1) |
| rs1555340  (CNR1) | 88922785 | 0.244  C | 0.00 | -0.05 - 0.06 | .926 | 547 | NONE |

Note: All genotypes were coded to reflect an additive model where -1 = common homozygote, 0 = heterozygote and 1 = rare homozygote

Regression weights (***β***) significantly less than 0 indicate that an increasing number of copies of the minor allele of the SNP was associated with a greater reduction in symptom severity across the active treatment or follow-up period. Values significantly greater than 0 indicate that an increasing number of copies of the minor allele of the SNP was associated with a poorer reduction in symptom severity.

^a^ n reflects total number of cases included in regression analysis for the sentinel SNP.

Table SV: Statistics for all clumps (identified by sentinel SNP) with treatment response between post-treatment and follow-up for the full sample

| **Sentinel SNP (Gene)** | **Clump BP** | **MAF/Minor allele** | **β** | **95% CI** | ***p*** | **n^a^** | **Additional SNPs included in clump**  **(SNPs with *p* values < .05 highlighted in bold, total number of additional SNPs in clump given in brackets e.g. (74))** |
| --- | --- | --- | --- | --- | --- | --- | --- |
|  |  |  |  |  |  |  |  |
| rs806365  (CNR1) | 88843390 - 88845949 | 0.408  T | 0.11 | 0.04 – 0.18 | .004 | 702 | rs10485171 (1) |
| rs2501431  (CNR2) | 24108683 - 24206032 | 0.423  G | 0.09 | 0.03 –0.16 | .007 | 874 | rs10917425, rs12031592, rs2256179, rs974698, rs1018396, rs2502986, rs6679378, rs2502987, rs2982390, rs2473377, rs2179395, rs2502979, rs2502980, rs34883557, rs6697805, rs6424115, rs60013677, rs34477640, rs7519554, rs35385477, rs6424116, rs71575777, rs10917430, exm31570, rs12748109, rs11803575, rs12755062, rs12141409, rs34570472, rs10799804, rs3123554, rs4483353, **rs2503002**, rs2503001, rs4649123, rs2503000, rs2502999, rs2502998, rs2502997, rs2501417, rs6680132, rs6672157, rs2501423, rs2502996, rs2502995, rs2501425, rs6663474, rs3003334, **rs35955796**, rs3003335, rs6665733, rs1130321, rs1130320, rs1106, rs1105, rs2229585, rs2229584, rs2229583, rs2229581, rs2229580, rs2502993, rs4649124, rs3003336, rs2502992, exm31668, rs2502991, rs6667839, rs6672499, rs2501433, rs6658703, rs2501434, rs6673210, **rs3123555**, **rs3123556**, rs6424119, rs6424120, rs4341315, rs2501367, rs2502990, **rs2502989**, rs2502988, **rs2501369** (82) |
| rs2070956  (CNR2) | 24191219 -24223859 | 0.101  C | 0.14 | 0.02 – 0.26 | .021 | 698 | rs12133557, **rs74937660**, **rs74223776**, **rs3003325**, **rs3003328**, **rs3003329**, rs3003621, rs3003332, **rs2070955**, **rs2070954, rs2070953**, **rs3003320**, **rs3003622**, **rs3003321**, **rs6664030, rs2503003**, **exm31621**, rs12754324, rs12727867, rs28404091, rs7541711, rs7541713, rs7541819, rs7532916, rs7541841, rs12741866, rs12759455, rs12733278, rs12759917, rs4625225, rs60390132, rs7519729, rs111834151, rs12742876, rs61778195, rs5026902, rs4648919, rs143281762, rs2501398, rs6424127, rs6424128, rs6424129, rs6424130, rs7512349, rs2502967, rs2501399, rs2501400, rs2502968, rs2501401, rs2502969, rs2502970, rs2501402, rs6424131, rs7415219, rs7550908, rs7550371, rs7537224, rs12730734, rs201557597, rs2501403, rs3003326, rs28735813, rs35829803, rs9424397, rs9424398, rs12724034, kgp1774784, rs2502971, rs2502972, rs9424400, rs9424338, rs2501404, rs3003327, rs3003623, rs2502973, rs2502974 (76) |
| rs6928813  (CNR1) | 88860482 - 88885426 | 0.180  G | -0.11 | -0.20 − -0.01 | .033 | 702 | **rs2023239**, rs1535255, rs806379, rs6928499, rs6454676, rs6912668, rs9450901, rs9450902, rs9444586, rs9450903, rs9450904, rs10485170, rs74471317, rs9450906, rs9444587, rs9450907, rs11966501, rs11968764, rs11963892, rs78335089, rs9444588, rs6454678 (22) |
| rs7769940  (CNR1) | 88947649 - 88973751 | 0.209  T | 0.10 | 0.01 – 0.19 | .034 | 702 | rs11966650, rs28816226, rs6910128, rs2325103, rs7753442, rs35750466, rs6922315, rs5878063, rs3929734, rs34367043, rs59413611, rs55845894, rs6918613, rs7765818, rs61310563, rs76763854, rs7762344, rs59838907, rs1408701, rs7453339, rs6454683, rs6454685, rs1408702 (23) |
| rs2209172  (FAAH) | 46938837 - 46978946 | 0.206  T | 0.09 | 0.00 – 0.18 | .044 | 702 | rs2031247, rs199790074, exm2268681, rs12132747, **rs4660353**, **rs56393814** (6) |
| rs74342198  (FAAH) | 46937668 - 46960043 | 0.019  A | 0.26 | -0.01 - 0.53 | 0.06 | 689 | rs74810435, rs76181455, rs56953705, rs60712134, rs112699995 (5) |
| rs2325100  (CNR1) | 88898611 - 88903557 | 0.271  G | -0.08 | -0.16-0.01 | 0.073 | 693 | rs9294397, rs9344758, rs12209554, rs9353529, rs6920617, rs35670824, rs719537, rs9351140, rs9362468, rs2325099 (10) |
| rs806371  (CNR1) | 88850150 - 88857320 | 0.107  G | -0.09 | -0.20 - 0.01 | 0.087 | 742 | rs806368 (1) |
| rs6678149  (FAAH) | 46761496 - 46888039 | 0.280  A | -0.07 | -0.15 - 0.01 | 0.089 | 694 | rs10890388, rs10890390, rs4660925, rs2145408, rs6429600, rs4141964, rs3766246, exm56619, rs324420, rs324418, rs2295633, rs12029329, rs201392030, rs61784641, rs55646923, rs4660928, rs6670926, rs4660346 (18) |
| rs140815810  (FAAH) | 46761389 - 46798466 | 0.010  I^b^ | 0.29 | -0.08 - 0.66 | 0.125 | 693 | rs6676038, rs7548226, rs144930516, rs142618742, rs7542864, rs7545120, rs200232349, rs17102133, rs114531159, rs199585121, rs112746922, rs112387082, rs147637102 (13) |
| rs1049353  (CNR1) | 88853635 | 0.284  A | -0.05 | -0.12 - 0.02 | 0.181 | 900 | NONE |
| rs1358791  (CNR1) | 88907388 - 88941764 | 0.244  T | -0.05 | -0.14-0.03 | 0.226 | 696 | rs9450916, rs16880378, rs2038447, rs12212677, rs7766691, rs9362470, rs1555340, rs9294398, rs1324958, rs1324957, rs9344763, rs9344764, rs9353531, rs9362472, rs9344765, rs9351142, rs12197767, rs9351143, rs9344767 (19) |
| rs6906154  (CNR1) | 88919215 - 88940323 | 0.053  T | 0.10 | -0.07-0.27 | 0.244 | 690 | rs7754491, rs7769918, rs56396859, rs58370001, rs9450925, rs59449423, rs55882449, rs60315037, rs113006189, rs1216678, rs16880396, rs145705030, rs7756920, rs7757556, rs62431489, rs6927294, rs62431491, rs964647, rs59903039 (19) |
| rs12213790  (CNR1) | 88898440 - 88902564 | 0.395  A | 0.04 | -0.03 - 0.12 | 0.259 | 702 | rs2038448, rs57809420, rs2325098 (3) |
| rs4110477  (FAAH) | 46766751 - 46865387 | 0.119  A | 0.06 | -0.05 - 0.18 | 0.287 | 694 | rs3753362, rs79783387, exm56515, rs77526109, rs76976909, rs76771627, exm56596, rs41294484, rs59082884, rs3891758, rs45480993, rs45540335, rs324416 (13) |
| rs12075550  (FAAH) | 46806242 - 46886953 | 0.400  C | 0.04 | -0.04 - 0.11 | 0.312 | 696 | rs67545510, rs112712935, rs41293273, rs41293275, rs17361749, rs41293277, rs10489769, rs11579255, rs72885163, rs68083747, rs66972124, rs17357621, rs56909107, rs41293285, rs17357628, rs17357635, rs68191463, rs41293287, rs56126529, rs7525309, rs7548675, rs7515284, rs41294456, rs66516678, rs41294458, rs79697925, exm56547, rs17361763, rs6684274, rs11304172, rs6696777, rs6659228, rs10661193, rs6697123, rs67487250, rs113610895, rs112280595, rs112491752, rs112089006, rs72677585, rs17357676, rs17361791, rs41294460, rs72885198, rs79490411, rs7556425, rs61358317, rs72886903, rs68112720, rs17361805, rs72677587, rs17361812, rs66575205, rs72886907, rs111226885, rs111699884, rs72886911, rs56344958, rs56121132, rs5013329, rs5013330, rs17357683, rs6667861, rs1057533, rs1057534, rs1057535, rs1135459, rs1135460, rs1475390, rs5773907, rs1475389, rs1475388, rs57267378, rs58162700, rs1812705, rs966907, rs112324418, rs72677588, rs72677589, rs66828173, rs72677590, rs72677591, rs72677592, rs41294476, rs41294478, rs17413701, rs7515598, rs7529674, rs12385696, rs12385697, rs12385693, rs12385695, rs28699008, rs56179746, rs146398931, rs6679080, rs6690075, rs6666601, rs6682266, rs34160166, rs6679898, rs10158236, rs10157084, rs112980938, rs56363851, rs66990604, rs201127808, rs10158572, rs10158130, rs10157464, rs2145409, rs56030283, rs55971480, rs66911505, rs6659448, rs6677394, rs6674726, rs11802866, rs112911951, rs113033705, rs12404971, rs72677593, rs72677594, rs7520482, rs7520497, rs7532149, rs28578741, rs202085913, rs55999016, rs56063031, rs67276232, rs12040179, rs6669025, rs200601367, rs55887761, rs9661240, rs1053624, rs1053627, rs1053628, rs6429599, rs17361819, rs67142569, rs17361833, rs41534051, rs17357711, rs6683192, rs10252, rs12062, rs111725921, rs66847432, rs67547686, rs113076306, rs17357759, rs17361873, rs72677596, rs72677599, rs72677600, rs17361887, rs55670684, rs56049453, rs67200518, rs141064494, rs72677602, rs10890391, rs67941619, rs66483119, rs55668511, rs55693298, rs11211270, rs68012736, rs143856001, rs11804189, rs144535648, rs147142636, rs56733721, rs66473412, rs6659681, rs6659788, rs55921163, rs55926300, rs111868160, rs3863641, rs12073998, rs13374893, rs13374968, rs6694628, rs6658556, rs6695043, rs72679807, rs56284503, rs17361915, rs113623605, rs11211271, rs144250006, rs113534835, rs56358525, rs6681857, rs56349187, rs79610407, rs72890715, rs4372193, rs111723629, rs3991877, rs35056299, rs57240150, rs113887859, rs201070992, rs61519400, rs56306849, rs913168, rs1984491, rs1984490, rs11589812, rs45449893, rs45517837, rs932816, rs72890727, rs6674305, rs56130131, rs11288511, rs6703374, rs6703669, rs45524035, rs17361936, rs17361950, rs11576941, rs6662982, rs55939860 (228) |
| rs2875545  (CNR1) | 88894422 - 88901540 | 0.195  G | 0.05 | -0.04-0.14 | 0.322 | 702 | rs1535257, rs7747006, rs7751075, rs16880345, rs1321361, rs9450914, rs9450915 (7) |
| rs806376 (CNR1) | 88858648 - 88867925 | 0.495  C | -0.04 | -0.11 - 0.04 | 0.326 | 702 | rs12205430 (1) |
| rs6698196  (FAAH) | 46975773 - 46975877 | 0.184  C | 0.04 | -0.06-0.13 | 0.468 | 695 | rs6683116 (1) |
| rs324425  (FAAH) | 46834173 - 46888905 | 0.058  T | -0.05 | -0.21-0.10 | 0.494 | 696 | rs324410, rs324424, rs324423, rs324421 (4) |
| rs9450876  (CNR1) | 88796053 - 88797885 | 0.344  A | -0.02 | -0.10 - 0.06 | 0.565 | 700 | rs9450877, rs1324075 (2) |
| rs3766248  (FAAH) | 46773488 | 0.020  A | -0.07 | -0.33 - 0.19 | 0.592 | 698 | NONE |
| rs7752742  (CNR1) | 88914904 | 0.462  C | 0.02 | -0.05 - 0.09 | 0.629 | 702 | NONE |
| rs806374  (CNR1) | 88857320 | 0.341  C | -0.02 | -0.09 - 0.06 | 0.698 | 702 | NONE |
| rs11211282  (FAAH) | 46905802 - 46928349 | 0.427  A | -0.01 | -0.09 - 0.06 | 0.729 | 702 | rs11211276, rs12407178, rs12409747, rs7538292, rs3795315, rs72637962, rs12126376, rs11211278, rs11293072, rs2281775, rs2281774, rs4660347, rs942258, rs10890397, rs4660933, rs2031248, rs10890398, rs4660348 (18) |
| rs4707441 (CNR1) | 88899850 - 88904498 | 0.144  G | -0.02 | -0.12-0.09 | 0.73 | 702 | rs9450913, rs4707442 (2) |
| rs7531088  (FAAH) | 46954587 - 46974497 | 0.177  C | 0.01 | -0.09-0.11 | 0.803 | 691 | rs1998545, rs1886117 (2) |
| rs6913146  (CNR1) | 88817588 - 88817934 | 0.123  T | 0.00 | -0.11-0.12 | 0.954 | 697 | rs6908693, rs6908755 (2) |
| rs2180619  (CNR1) | 88877952 | 0.401  G | 0.00 | -0.07-0.06 | 0.978 | 875 | NONE |

Note: All genotypes were coded to reflect an additive model where -1 = common homozygote, 0 = heterozygote and 1 = rare homozygote

Regression weights (***β***) significantly less than 0 indicate that an increasing number of copies of the minor allele of the SNP was associated with a greater reduction in symptom severity across the active treatment or follow-up period. Values significantly greater than 0 indicate that an increasing number of copies of the minor allele of the SNP was associated with a poorer reduction in symptom severity.

^a^ n reflects total number of cases included in regression analysis for the sentinel SNP.

^b^ Insertion is TATTCACATG

Table SVI: Statistics for all clumps (identified by sentinel SNP) with treatment response between post-treatment and follow-up in the subset of the sample with fear-based anxiety disorder diagnoses

| **Sentinel SNP (Gene)** | **Clump BP** | **MAF/Minor allele** | **β** | **95% CI** | ***p*** | **n^a^** | **Additional SNPs included in clump**  **(SNPs with *p* values < .05 highlighted in bold, total number of additional SNPs in clump given in brackets e.g. (74))** |
| --- | --- | --- | --- | --- | --- | --- | --- |
|  |  |  |  |  |  |  |  |
| rs806365  (CNR1) | 88843390 - 88845949 | 0.392  T | 0.17 | 0.07 – 0.27 | .**001** | 399 | rs10485171 (1) |
| rs7769940  (CNR1) | 88947649 - 88973751 | 0.216  T | 0.19 | 0.07 – 0.32 | .003 | 399 | **rs11966650**, **rs28816226**, **rs6910128**, **rs2325103**, **rs7753442**, **rs35750466**, **rs6922315**, **rs5878063**, **rs3929734**, **rs34367043**, **rs59413611**, **rs55845894**, **rs6918613**, **rs7765818**, **rs61310563**, **rs76763854**, **rs7762344**, **rs59838907**, **rs1408701**, **rs7453339**, **rs6454683**, **rs6454685**, rs1408702 (23) |
| rs2501431  (CNR2) | 24108683 – 24206032 | 0.448  G | 0.14 | 0.04 –0.23 | .004 | 495 | rs10917425, rs12031592, rs2256179, rs974698, rs1018396, rs2502986, rs6679378, rs2502987, **rs2982390**, **rs2473377**, rs2179395, **rs2502979**, **rs2502980**, **rs34883557**, **rs6697805**, **rs6424115**, **rs60013677**, **rs34477640**, **rs7519554**, rs35385477, rs6424116, rs71575777, rs10917430, exm31570, rs12748109, rs11803575, rs12755062, rs12141409, rs34570472, rs10799804, **rs3123554**, **rs4483353**, r**s2503002**, **rs2503001**, **rs4649123**, **rs2503000**, **rs2502999**, **rs2502998**, **rs2502997**, **rs2501417**, **rs6680132**, **rs6672157**, **rs2501423**, **rs2502996**, **rs2502995**, **rs2501425**, **rs6663474**, **rs3003334**, **rs35955796**, **rs3003335,** **rs6665733**, **rs1130321**, **rs1130320**, **rs1106**, **rs1105**, **rs2229585**, **rs2229584**, **rs2229583**, **rs2229581**, **rs2229580**, **rs2502993**, **rs4649124**, **rs3003336**, **rs2502992**, **exm31668**, **rs2502991**, **rs6667839**, **rs6672499**, **rs2501433**, **rs6658703**, r**s2501434**, **rs6673210**, **rs3123555**, **rs3123556**, **rs6424119**, **rs6424120**, **rs4341315**, **rs2501367**, **rs2502990**, **rs2502989**, **rs2502988**, **rs2501369** (82) |
| rs1049353  (CNR1) | 88853635 | 0.282  A | -0.09 | -0.19 - 0.01 | 0.067 | 512 | NONE |
| exm2268681  (FAAH) | 46938837 – 46978946 | 0.391  C | 0.1 | -0.01 - 0.20 | 0.070 | 399 | rs2031247, rs199790074, rs12132747, rs4660353, rs2209172, rs56393814 (6) |
| rs74342198  (FAAH) | 46937668 - 46960043 | 0.020  A | 0.31 | -0.04 - 0.67 | 0.084 | 393 | rs74810435, rs76181455, rs56953705, rs60712134, rs112699995 (5) |
| rs2070954  (CNR2) | 24191219 - 24223859 | 0.101  G | 0.14 | -0.03-0.30 | 0.103 | 398 | rs12133557, rs74937660, rs74223776, rs3003325, rs3003328, rs3003329, rs3003621, rs3003332, rs2070956, rs2070955, rs2070953, rs3003320, rs3003622, rs3003321, rs6664030, rs2503003, exm31621, rs12754324, rs12727867, rs28404091, rs7541711, rs7541713, rs7541819, rs7532916, rs7541841, rs12741866, rs12759455, rs12733278, rs12759917, rs4625225, rs60390132, rs7519729, rs111834151, rs12742876, rs61778195, rs5026902, rs4648919, rs143281762, rs2501398, rs6424127, rs6424128, rs6424129, rs6424130, rs7512349, rs2502967, rs2501399, rs2501400, rs2502968, rs2501401, rs2502969, rs2502970, rs2501402, rs6424131, rs7415219, rs7550908, rs7550371, rs7537224, rs12730734, rs201557597, rs2501403, rs3003326, rs28735813, rs35829803, rs9424397, rs9424398, rs12724034, kgp1774784, rs2502971, rs2502972, rs9424400, rs9424338, rs2501404, rs3003327, rs3003623, rs2502973, rs2502974 (76) |
| rs2325100  (CNR1) | 88898611 - 88903557 | 0.280  G | -0.1 | -0.22-0.02 | 0.110 | 393 | rs9294397, rs9344758, rs12209554, rs9353529, rs6920617, rs35670824, rs719537, rs9351140, rs9362468, rs2325099 (10) |
| rs806368  (CNR1) | 88850150 - 88857320 | 0.217  G | -0.08 | -0.18-0.02 | 0.128 | 520 | rs806371, rs806374 (2) |
| rs4110477  (FAAH) | 46766751 - 46865387 | 0.129  A | 0.11 | -0.04 - 0.27 | 0.143 | 395 | rs3753362, rs79783387, exm56515, rs77526109, rs76976909, rs76771627, exm56596, rs41294484, rs59082884, rs3891758, rs45480993, rs45540335, rs324416 (13) |
| exm56547  (FAAH) | 46806959 - 46882753 | 0.298  G | 0.08 | -0.03-0.18 | 0.164 | 399 | rs11579255, rs34160166, rs2145409, rs11802866, rs12404971, rs12040179, rs10252, rs12075550, rs141064494, rs10890390, rs10890391, rs11211270, rs11804189, rs56733721, rs3863641, rs12073998, rs11211271, rs4660925, rs35056299, rs913168, rs11589812, rs4141964, rs3766246, rs2295633, rs11576941, rs61784641, rs55646923 (27) |
| rs6678149  (FAAH) | 46761496 - 46888039 | 0.282  A | -0.08 | -0.20 - 0.04 | 0.173 | 393 | rs10890388, rs2145408, rs6429600, exm56619, rs324420, rs324418, rs12029329, rs201392030, rs4660928, rs6670926, rs4660346 (11) |
| rs10890398  (FAAH) | 46925594 - 46928349 | 0.173  C | 0.09 | -0.04 - 0.23 | 0.181 | 399 | rs4660348, rs11211282 (2) |
| rs1358791  (CNR1) | 88907388 - 88941764 | 0.244  T | -0.07 | -0.19-0.04 | 0.211 | 393 | rs9450916, rs16880378, rs2038447, rs12212677, rs7766691, rs9362470, rs1555340, rs9294398, rs1324958, rs1324957, rs9344763, rs9344764, rs9353531, rs9362472, rs9344765, rs9351142, rs12197767, rs9351143, rs9344767 (19) |
| rs61358317  (FAAH) | 46806242 - 46886953 | 0.280  T | -0.07 | -0.19-0.04 | 0.216 | 396 | rs67545510, rs112712935, rs41293273, rs41293275, rs17361749, rs41293277, rs10489769, rs72885163, rs68083747, rs66972124, rs17357621, rs56909107, rs41293285, rs17357628, rs17357635, rs68191463, rs41293287, rs56126529, rs7525309, rs7548675, rs7515284, rs41294456, rs66516678, rs41294458, rs79697925, rs17361763, rs6684274, rs11304172, rs6696777, rs6659228, rs10661193, rs6697123, rs67487250, rs113610895, rs112280595, rs112491752, rs112089006, rs72677585, rs17357676, rs17361791, rs41294460, rs72885198, rs79490411, rs7556425, rs72886903, rs68112720, rs17361805, rs72677587, rs17361812, rs66575205, rs72886907, rs111226885, rs111699884, rs72886911, rs56344958, rs56121132, rs5013329, rs5013330, rs17357683, rs6667861, rs1057533, rs1057534, rs1057535, rs1135459, rs1135460, rs1475390, rs5773907, rs1475389, rs1475388, rs57267378, rs58162700, rs1812705, rs966907, rs112324418, rs72677588, rs72677589, rs66828173, rs72677590, rs72677591, rs72677592, rs41294476, rs41294478, rs17413701, rs7515598, rs7529674, rs12385696, rs12385697, rs12385693, rs12385695, rs28699008, rs56179746, rs146398931, rs6679080, rs6690075, rs6666601, rs6682266, rs6679898, rs10158236, rs10157084, rs112980938, rs56363851, rs66990604, rs201127808, rs10158572, rs10158130, rs10157464, rs56030283, rs55971480, rs66911505, rs6659448, rs6677394, rs6674726, rs112911951, rs113033705, rs72677593, rs72677594, rs7520482, rs7520497, rs7532149, rs28578741, rs202085913, rs55999016, rs56063031, rs67276232, rs6669025, rs200601367, rs55887761, rs9661240, rs1053624, rs1053627, rs1053628, rs6429599, rs17361819, rs67142569, rs17361833, rs41534051, rs17357711, rs6683192, rs12062, rs111725921, rs66847432, rs67547686, rs113076306, rs17357759, rs17361873, rs72677596, rs72677599, rs72677600, rs17361887, rs55670684, rs56049453, rs67200518, rs72677602, rs67941619, rs66483119, rs55668511, rs55693298, rs68012736, rs143856001, rs144535648, rs147142636, rs66473412, rs6659681, rs6659788, rs55921163, rs55926300, rs111868160, rs13374893, rs13374968, rs6694628, rs6658556, rs6695043, rs72679807, rs56284503, rs17361915, rs113623605, rs144250006, rs113534835, rs56358525, rs6681857, rs56349187, rs79610407, rs72890715, rs4372193, rs111723629, rs3991877, rs57240150, rs113887859, rs201070992, rs61519400, rs56306849, rs1984491, rs1984490, rs45449893, rs45517837, rs932816, rs72890727, rs6674305, rs56130131, rs11288511, rs6703374, rs6703669, rs45524035, rs17361936, rs17361950, rs6662982, rs55939860 (207) |
| rs1886117  (FAAH) | 46954587 - 46974497 | 0.313  T | 0.06 | -0.05-0.17 | 0.290 | 398 | rs1998545, rs7531088 (2) |
| rs16880345  (CNR1) | 88894422 - 88901540 | 0.191  G | 0.06 | -0.06 - 0.19 | 0.333 | 396 | rs1535257, rs7747006, rs7751075, rs2875545, rs1321361, rs9450914, rs9450915 (7) |
| rs9450913  (CNR1) | 88899850 - 88904498 | 0.134  T | 0.06 | -0.08-0.21 | 0.393 | 392 | rs4707441, rs4707442 (2) |
| rs12205430  (CNR1) | 88858648 - 88867925 | 0.196  C | 0.05 | -0.07 - 0.18 | 0.394 | 399 | rs806376, rs806379 (2) |
| rs1535255  (CNR1) | 88860482 - 88885426 | 0.158  G | -0.05 | -0.18 - 0.07 | 0.420 | 492 | rs2023239, rs6928499, rs6928813, rs6454676, rs6912668, rs9450901, rs9450902, rs9444586, rs9450903, rs9450904, rs10485170, rs74471317, rs9450906, rs9444587, rs9450907, rs11966501, rs11968764, rs11963892, rs78335089, rs9444588, rs6454678 (21) |
| rs7752742  (CNR1) | 88914904 | 0.454  C | 0.04 | -0.06 - 0.14 | 0.422 | 399 | NONE |
| rs9450876  (CNR1) | 88796053 - 88797885 | 0.364  A | -0.04 | -0.15 - 0.07 | 0.481 | 398 | rs9450877, rs1324075 (2) |
| rs200232349  (FAAH) | 46761389 - 46798466 | 0.011  I^b^ | -0.16 | -0.63-0.32 | 0.520 | 394 | rs6676038, rs7548226, rs144930516, rs142618742, rs7542864, rs7545120, rs17102133, rs114531159, rs140815810, rs199585121, rs112746922, rs112387082, rs147637102 (13) |
| rs324424  (FAAH) | 46834173 - 46888905 | 0.059  A | -0.07 | -0.28 - 0.15 | 0.553 | 396 | rs324410, rs324425, rs324423, rs324421 (4) |
| rs6908755  (CNR1) | 88817588 - 88817934 | 0.123  T | 0.04 | -0.11-0.20 | 0.577 | 399 | rs6908693, rs6913146 (2) |
| rs58370001  (CNR1) | 88919215 - 88940323 | 0.043  C | 0.07 | -0.18 - 0.32 | 0.589 | 392 | rs7754491, rs7769918, rs56396859, rs9450925, rs59449423, rs55882449, rs60315037, rs113006189, rs1216678, rs16880396, rs145705030, rs7756920, rs7757556, rs62431489, rs6927294, rs62431491, rs964647, rs59903039, rs6906154 (19) |
| rs6698196  (FAAH) | 46975773 - 46975877 | 0.184  C | 0.03 | -0.10 - 0.17 | 0.635 | 395 | rs6683116 (1) |
| rs11211276  (FAAH) | 46905802 - 46923102 | 0.232  T | 0.02 | -0.10-0.14 | 0.775 | 399 | rs12407178, rs12409747, rs7538292, rs3795315, rs72637962, rs12126376, rs11211278, rs11293072, rs2281775, rs2281774, rs4660347, rs942258, rs10890397, rs4660933, rs2031248 (15) |
| rs12213790  (CNR1) | 88898440 - 88902564 | 0.390  A | 0.01 | -0.09 - 0.11 | 0.825 | 399 | rs2038448, rs57809420, rs2325098 (3) |
| rs3766248  (FAAH) | 46773488 | 0.018  A | 0.02 | -0.34 - 0.39 | 0.900 | 396 | NONE |
| rs2180619  (CNR1) | 88877952 | 0.413  G | 0.00 | -0.09-0.09 | 0.997 | 494 | NONE |

Note: All genotypes were coded to reflect an additive model where -1 = common homozygote, 0 = heterozygote and 1 = rare homozygote

Regression weights (***β***) significantly less than 0 indicate that an increasing number of copies of the minor allele of the SNP was associated with a greater reduction in symptom severity across the active treatment or follow-up period. Values significantly greater than 0 indicate that an increasing number of copies of the minor allele of the SNP was associated with a poorer reduction in symptom severity.

^a^ n reflects total number of cases included in regression analysis for the sentinel SNP.

^b^ Insertion is CA

**References**

Barrett PM. 2004. FRIENDS for Life program - Group leader's workbook for children. Brisbane, Queensland: Australian Academic Press.

Barrett PM, Farrell LJ, Ollendick TH, Dadds M. 2006. Long-Term Outcomes of an Australian Universal Prevention Trial of Anxiety and Depression Symptoms in Children and Youth: An Evaluation of the Friends Program. Journal of Clinical Child & Adolescent Psychology 35(3):403-411.

Bögels SM. 2008. Behandeling van angststoornissen bij kinderen en adolescenten. Springer.

Creswell C, Cruddace S, Gerry S, Gitau R, McIntosh E, Mollison J, Murray L, Shafran R, Stein A, Violato M, Voysey M, Willetts L, Williams N, Yu LM, Cooper PJ. 2015. Treatment of childhood anxiety disorder in the context of maternal anxiety disorder: a randomised controlled trial and economic analysis. Health Technol Assess 19(38).

Creswell C, Hentges F, Parkinson M, Sheffield P, Willetts L, Cooper P. 2010. Feasibility of guided cognitive behaviour therapy (CBT) self-help for childhood anxiety disorders in primary care. Mental Health in Family Medicine 7(1):49-57.

Hiller R, Apetroaia A, Clarke K, Hughes Z, Orchard F, Parkinson P, Creswell C. In Submission. The Effect of Targeting Tolerance of Children's Negative Emotions among Anxious Parents of Children with Anxiety Disorders: A Pilot Randomised Controlled Trial.

Hudson JL, Newall C, Rapee RM, Lyneham HJ, Schniering CC, Wuthrich VM, Schneider S, Seeley-Wait E, Edwards S, Gar NS. 2013. The Impact of Brief Parental Anxiety Management on Child Anxiety Treatment Outcomes: A Controlled Trial. Journal of Clinical Child & Adolescent Psychology 1-11.

Kendall PC. 1994. Treating anxiety disorders in children: Results of a randomized clinical trial. J Consult Clin Psychol 62(1):100-110.

Lyneham HJ, Rapee RM. 2006. Evaluation of therapist-supported parent-implemented CBT for anxiety disorders in rural children. Behav Res Ther 44(9):1287-1300.

Nauta MH, Scholing A. 1998. Cognitieve gedragstherapie bij kinderen en jongeren met angststoornissen: een protocol van 12 sessies. Handleiding voor de therapeut. Groningen: Rijksuniversiteit Groningen (Klinische en Ontwikkelingspsychologie).

Orchard F, Apetroaia A, Clarke K, Hirsch C, Creswell C. In Submission. Cognitive Bias Modification of Interpretation in Children with Social Anxiety Disorder.

Rapee R, Lyneham H, Schniering C, Wuthrich V, Abbott M, Hudson J, Wignall A. 2006a. The Cool Kids® Child and Adolescent Anxiety Program. Sydney: Centre for Emotional Health, Macquarie University.

Rapee RM, Abbott MJ, Lyneham HJ. 2006b. Bibliotherapy for children with anxiety disorders using written materials for parents: A randomized controlled trial. J Consult Clin Psychol 74(3):436-444.

Rapee RM, Lyneham HJ, Schniering CA, Wuthrich VM, Abbott MJ, Hudson JL, Wignall A. 2006c. Cool Kids "Chilled" Adolescent Anxiety Program. Sydney: MUARU, Macquarie University.

Schneider S, Blatter-Meunier J, Herren C, In-Albon T, Adornetto C, Meyer A, Lavallee KL. 2013a. The efficacy of a family-based cognitive-behavioral treatment for separation anxiety disorder in children aged 8–13: A randomized comparison with a general anxiety program. Journal of Consulting and Clinical Psychology 81(5):932-940.

Schneider S, Lavallee K. 2013b. Separation Anxiety Disorder. In: C.A. E, T. O, editors. The Wiley-Blackwell Handbook of The Treatment of Childhood and Adolescent Anxiety: Wiley-Blackwell. p 301-334.

Smith P, Yule W, Perrin S, Tranah T, Dalgleish TIM, Clark DM. 2007. Cognitive-Behavioral Therapy for PTSD in Children and Adolescents: A Preliminary Randomized Controlled Trial. Journal of the American Academy of Child & Adolescent Psychiatry 46(8):1051-1061.

Spence SH, Donovan CL, March S, Gamble A, Anderson RE, Prosser S, Kenardy J. 2011. A randomized controlled trial of online versus clinic-based CBT for adolescent anxiety. Journal of consulting and clinical psychology 79(5):629.

Thirlwall K, Cooper PJ, Karalus J, Voysey M, Willetts L, Creswell C. 2013. Treatment of child anxiety disorders via guided parent-delivered cognitive-behavioural therapy: randomised controlled trial. British Journal of Psychiatry 203(6):436-444.

Vassilopoulos SP, Banerjee R, Prantzalou C. 2009. Experimental modification of interpretation bias in socially anxious children: Changes in interpretation, anticipated interpersonal anxiety, and social anxiety symptoms. Behaviour Research and Therapy 47(12):1085-1089.

Wergeland GJH, Fjermestad KW, Marin CE, Haugland BS-M, Bjaastad JF, Oeding K, Bjelland I, Silverman WK, Öst L-G, Havik OE, Heiervang ER. 2014. An effectiveness study of individual vs. group cognitive behavioral therapy for anxiety disorders in youth. Behaviour Research and Therapy 57(0):1-12.
